# Analysis of Single Locus Trajectories for Extracting In Vivo Chromatin Tethering Interactions

**DOI:** 10.1371/journal.pcbi.1004433

**Published:** 2015-08-28

**Authors:** Assaf Amitai, Mathias Toulouze, Karine Dubrana, David Holcman

**Affiliations:** 1 Institute for Medical Engineering & Science, The Massachusetts Institute of Technology (MIT), Cambridge, Massachusetts, United States of America; 2 Laboratory of genetic instability and nuclear organization, CEA, Fontenay-aux-Roses, France; 3 IBENS, Ecole Normale Supérieure, Paris, France and Mathematical Institute, University of Oxford, Oxford, United Kingdom; Rutgers University, UNITED STATES

## Abstract

Is it possible to extract tethering forces applied on chromatin from the statistics of a single locus trajectories imaged *in vivo*? Chromatin fragments interact with many partners such as the nuclear membrane, other chromosomes or nuclear bodies, but the resulting forces cannot be directly measured in vivo. However, they impact chromatin dynamics and should be reflected in particular in the motion of a single locus. We present here a method based on polymer models and statistics of single trajectories to extract the force characteristics and in particular when they are generated by the gradient of a quadratic potential well. Using numerical simulations of a Rouse polymer and live cell imaging of the MAT-locus located on the yeast *Saccharomyces cerevisiae* chromosome III, we recover the amplitude and the distance between the observed and the interacting monomer. To conclude, the confined trajectories we observed *in vivo* reflect local interaction on chromatin.

## Author Summary

Is it possible to recover the local environment, the external and internal forces acting on a polymer from a single locus trajectories? To study this question, we resolve this reverse cell biology problem by developing a method that uses *in vivo* live single locus trajectories to extract physical forces applied on chromatin. We applied the method to the statistics of the *S. cerevisiae* MAT-locus motion and recover tethering forces acting on the chromatin. The local confinement of a chromatin locus can either be due to crowding or to local interactions with partners such as the surface of the nuclear membrane, other chromosomes or nuclear bodies that cannot be directly measured. We conclude here that confined trajectories of a single chromatin locus can be generated by local tethering interactions. This approach is applicable to cells under various conditions, such as during double-stranded DNA break repair.

## Introduction

What can we learn about the local environment, the external and internal forces and the chromatin itself from the motion of a chromatin locus? This motion can be driven by local diffusion and/or forces between monomers of the model polymer [1–3]. Monomers motion is highly correlated due the polymer hierarchy of relaxation times [4, 5], leading in particular to anomalous diffusion [6, 7]. This anomalous behavior is well documented for chromatin loci [8–10] and we propose here to examine the effect of local external interactions on a locus motion. Much of the chromatin dynamics is reflected in the motion of a single chromosomal locus and conversely, a locus motion allows probing the chromatin dynamics [11, 12] at tens of nanometers and millisecond scales resolution [13–15]. When this motion is described as a free or confined Brownian motion, classical statistical tools such as the mean square displacement (MSD) and radius of confinement [16–18] can be used to extract the values of physical parameters. Other methods have been developed to extract kinetic rates about molecular events from forces imposed in pulling experiments [19, 20] or in the context of atomic force microscopy [21, 22].

**Fig 1:**
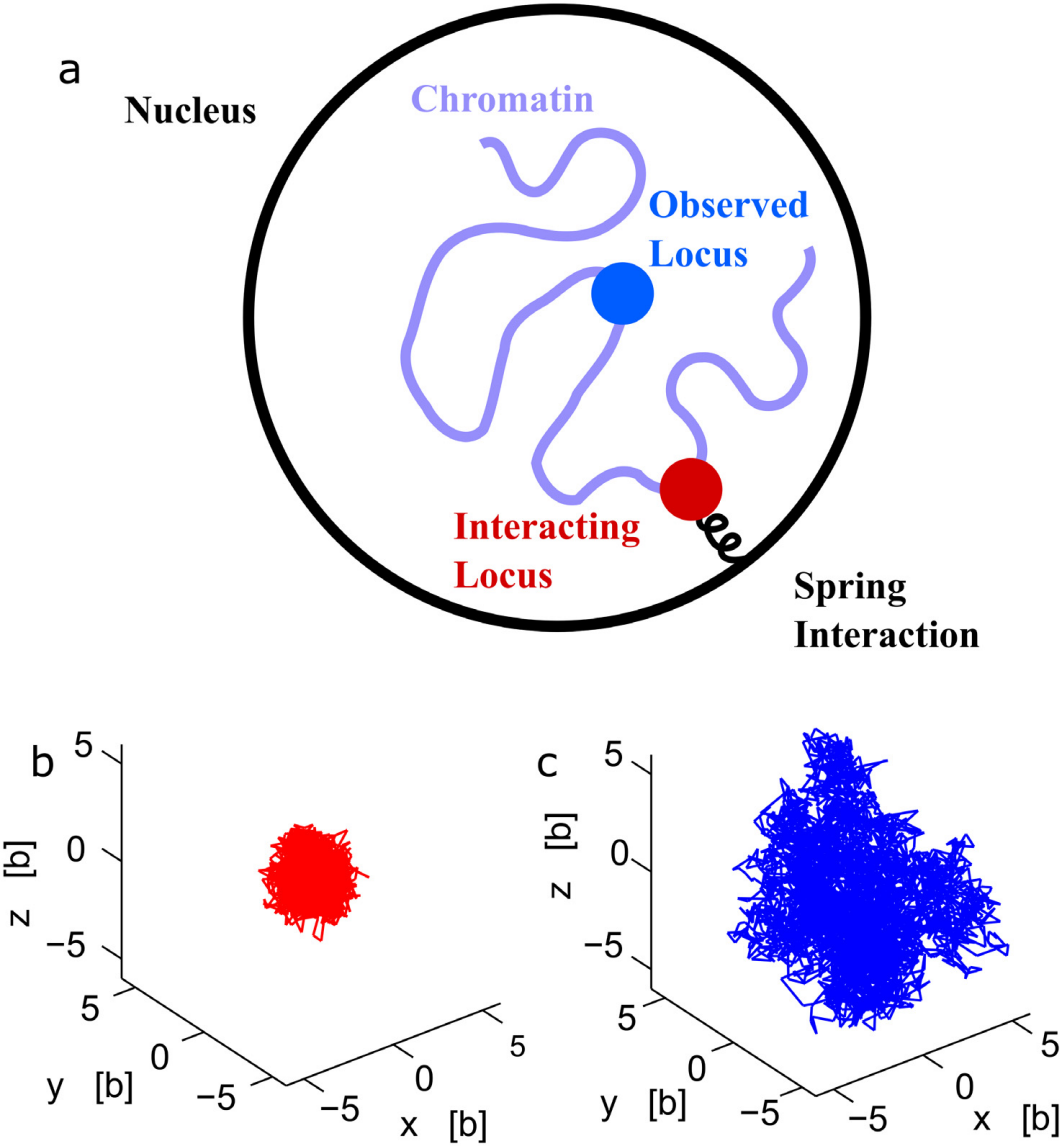
A polymer interacting with multiple potential wells. (a) Schematic representation of a polymer, where some monomers (red) interact with fixed harmonic potential wells, while monomer *c* (blue) is observed. (b-c) Stochastic trajectories of three monomers, part of a polymer, where the two extremities interact with two potential wells fixed at the origin and at position ***μ*** = (5*b*, 0, 0) respectively. The middle monomer trajectory (blue) is more extended than the two others, as shown for a polymer of length N = 21 (b) and N = 41 (c).

**Fig 2:**
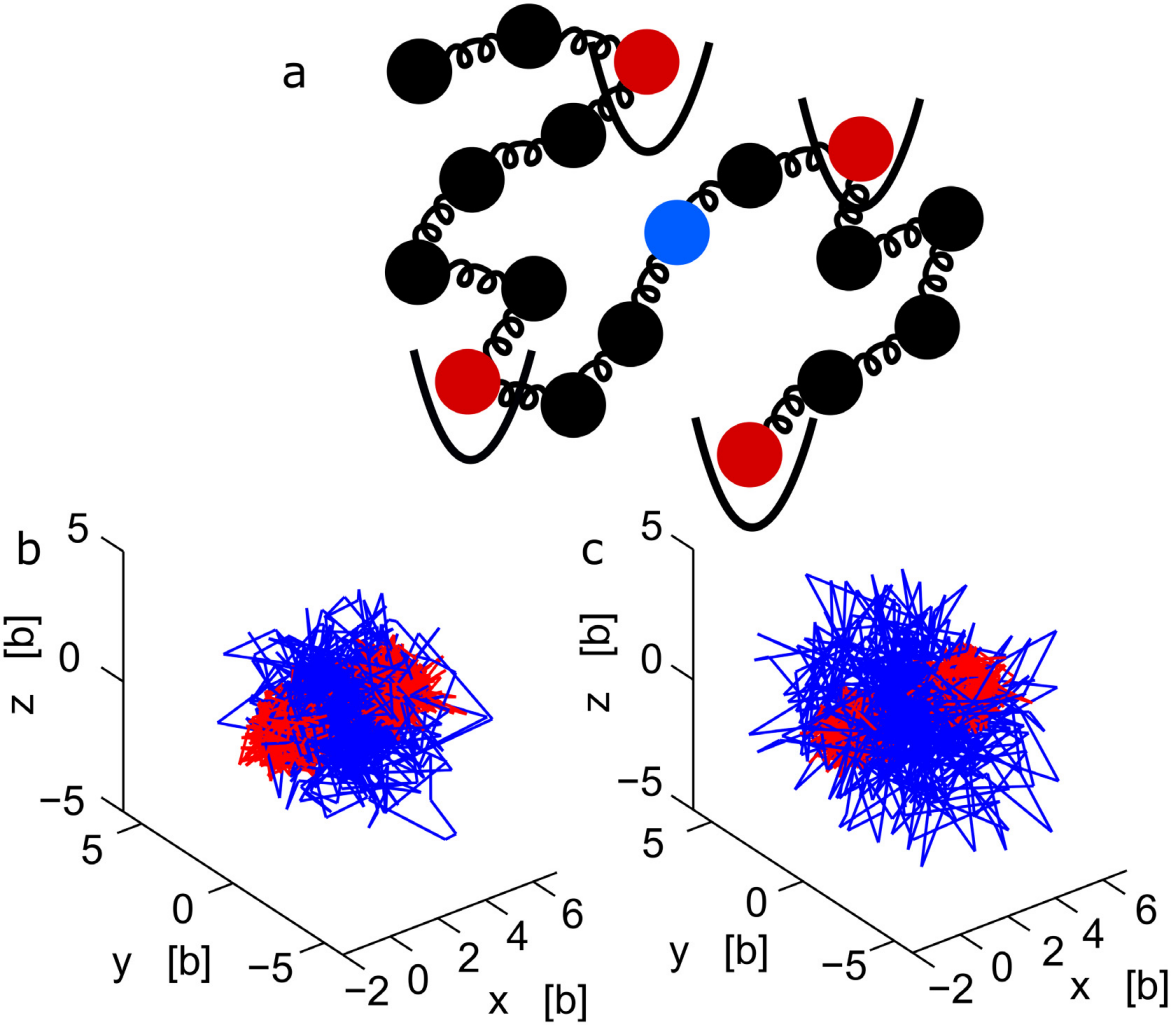
Dynamics of interacting versus observed locus. (**a**) Schematic representation of the nucleus, where one locus is observed and followed with a florescent label while another (non-visible) chromatin locus is interacting with another nuclear element. (**b-c**) Stochastic trajectories of monomers, part of a polymer (*N* = 30) where one extremity interacts with a harmonic potential well of strength *κ* = 2*K*_*B*_ *T*/*b* ^2^. When the observed monomer is the interacting monomer (red), the trajectory is well localized (b). When the middle monomer of the polymer is tracked, the trajectory (blue) is more extended (c).

Polymer models can account for various forces acting on chromatin, such as bending elasticity, internal rigidity, torsion and Lennard-Jones interactions [2]. In addition, the chromatin fiber can experience local fluctuations driven by ATP [23, 24], identified by micrometer long-range coherent [25] and active motion [26]. Other interactions can be due to repulsive forces or self-avoiding interactions with other chromatin parts, attractive forces driven by anchoring a locus at a nuclear pore [27] or tethering to the spindle pole body through the centromere [28] or with other chromosomes mediated by protein-protein interactions. While these interactions are local and extend to tens or hundreds nanometers, they can influence the polymer dynamics and in particular on this polymer, even if positioned far away from the interacting site (Fig 1a). We present here a method based on polymer models and statistical analysis of single particle trajectories, to estimate the local interactions acting on chromatin (Fig 2a). A sufficiently large ensemble of single tagged locus trajectories is the key ingredient of the method. When applied forces are stationary over the time course of the trajectory recording, we extract interactions or their mirror deterministic forces by deriving formulas that link the empirical velocity distribution of a locus to forces applied to a distant single monomer. The present method allows distinguishing external forces applied on a single monomer from intrinsic forces acting on monomers. The principle and the difficulty of the method can be understood as follows: for a single stochastic particle modeled by the Smoluchowski’s limit of the Langevin equation, the velocity of the particle **v** is proportional to a force **f** applied on the particle plus an additional white noise, summarized as

$$\begin{array}{c} \gamma v=f+\gamma \sqrt{2D}\dot{w}, \end{array}$$ (1)

where *γ* is the friction coefficient, *D* the diffusion coefficient and *w* is the normalized Wiener process. Thus by averaging over the ensemble of velocity realizations, it is possible to recover the first moment, which is the force field [29]. However, for a polymer chain, there are internal forces between monomers and thus, the difficulty that needs to be resolved here, as the data are measured at a single monomer, is to separate the internal forces acting on the measured monomer from the external ones acting on a monomer further away. This problem is resolved here, but the inversion formula to recover the force depends on the polymer model. When the external applied force is the gradient of a quadratic potential (second inversion formula) we explicit the formula analytically and show that the motion of the observed monomer is characterized by an effective force, with an effective elastic spring constant *k*_*c*_ that we compute. We simulate a Rouse polymer [4], which serves as a model for the chromatin structure [8, 30]. The locus motion cannot simply be approximated as an Ornstein-Uhlenbeck (OU) process, with an effective harmonic potential well, but we show that the effective force acting on the observed monomer decays with the distance along the chain between the interacting and the observed monomer. The effective spring constant *k*_*c*_ decays slower with this distance for a general class of polymers (*β*–polymer [31]) compared to Rouse. Applying the present approach to live cell-imaging data of the MAT-locus in yeast [32], which appears to be constrained shows that confined trajectories can either be due to local crowding or to direct interactions. Using Single Particle Trajectories (SPTs), we extract forces acting on that locus and show that trajectory localization is mediated by direct forces. This result validates the model predictions and the relation between the strength of a force applied on the chromatin locus and the radius of confinement. We conclude that local forces and not only crowding do confine chromatin motion. The present approach can further be applied to other situations, such as yeast telomeres anchored to the nuclear periphery [32], changes in single locus dynamics or repositioning following the induction of double-stranded DNA breaks.

## Results

### Polymer framework

When an external force, which is the gradient of the potential *U*_ext_(***R***) is applied to a Rouse polymer, the interaction is described by the energy

$$\begin{array}{c} \phi \left(R\right)=\frac{\kappa }{2}\sum_{j=2}^{N}{\left(R_{j}-R_{j-1}\right)}^{2}+U_{\text{ext}}(R), \end{array}$$ (2)

where ***R*** = (***R***_1_, ***R***_2_, …, ***R***_*N*_) is the ensemble of monomers, connected by a spring of strength *κ* = *dk*_*B*_ *T*/*b* ^2^. *b* is the standard-deviation of the distance between adjacent monomers [4], *k*_*B*_ the Boltzmann coefficient, *T* the temperature and *d* the dimensionality (dim 2 or 3). In the Smoluchowski’s limit of the Langevin equation [33], the dynamics of monomer ***R***_*n*_ is described by

$$\begin{array}{c} \frac{dR_{j}}{dt}=-D\nabla_{R_{j}}\phi \left(R\right)+\sqrt{2D}\frac{dw_{j}}{dt}, \end{array}$$ (3)

for *j* = 1, … *N* and each ***w***_*j*_ is an independent *d*-dimensional white noise with mean zero and variance 1, *D* is the monomer diffusion coefficient. We will describe specifically the field of ∇_***R*_*j*_**_ *U*_ext_(***R***) in the next subsection.

When the chromatin motion is described by Rouse chain, the effective diffusion coefficient can be estimated from data. We shall choose a reference monomer ***R***_*c*_, which represents the tagged locus. One of the key results of the present analysis is the following formula, which links the velocity or first moment of the monomer of ***R***_*c*_ (averaged over all realizations) to the polymer configuration distribution:

**General inversion formula:**

$$\begin{array}{c} \lim_{\Delta t\to 0}𝔼\left\{\frac{R_{c}\left(t+\Delta t\right)-R_{c}\left(t\right)}{\Delta t}|R_{c}=x\right\}=-D\int_{\Omega }dR_{1}..\int_{\Omega }dR_{N}\left(\nabla_{R_{c}}\phi \right)P\left(R|R_{c}=x\right), \end{array}$$ (4)

where 𝔼{.∣***R***_*c*_ = ***x***} denotes ensemble averaging under the condition that the tagged monomer is at position ***R***_*c*_ = ***x***. Formula 4 is generic and does not depend on the particular expression of the external forces acting on the polymer. Moreover, we do not impose here any restriction on the domain Ω where the polymer evolves. The polymer is reflected on the boundary ∂Ω. The conditional probability *P*(***R***∣***R***_*c*_ = ***x***) is computed from equilibrium probability distribution function (pdf) *P*(***R***_1_, ***R***_2_, …, ***R***_*N*_), which satisfies the Fokker-Planck equation (FPE) in the phase space Ω × ..Ω ⊂ ℝ^3*N*^,

$$\begin{array}{c} 0=\Delta P\left(R\right)+\nabla \cdot (\nabla \phi \;P(R)), \end{array}$$ (5)

with boundary condition

$$\begin{array}{c} \phi \frac{\partial P}{\partial n_{i}}+P\frac{\partial \phi }{\partial n_{i}}=0\;\text{for}\;R_{i}\in \partial \Omega \;\;\;\text{for}\;i=1..N, \end{array}$$

where ***n***_*i*_ is the normal vector to the boundary ∂Ω at position ***R***_*i*_.

### The external field of forces −∇*U*_ext_(*R*)

A permanent force located at position ***μ*** can be approximated at order two by a harmonic well. We suppose that this force is applied to monomer *n*. The force applied on ***R***_*n*_ is the gradient of the harmonic potential (Fig 1a)

$$\begin{array}{c} U_{\text{ext}}\left(R_{n}\right)=\frac{1}{2}k{\left(\mu -R_{n}\right)}^{2}, \end{array}$$ (6)

where *k* is the force constant. The monomer *n* that experiences the force is different from the tagged monomer *c* and we shall assume that *n* < *c*. As we shall see now, this potential well affects the dynamics of the entire polymer and specifically the observed locus *c*.

### Extracting an applied force from the ensemble of an observed monomer

To extract the strength of the potential well applied on monomer *n*, from the measured velocity of locus *c*, we derive an analytical expression for formula 4. First, the force acting on monomer *c*, when its position is ***x*** is given by

$$\begin{array}{ccc} F_{R_{c}=x}^{c} & = & -\nabla_{R_{c}}\phi {\left(R_{c-1},R_{c},R_{c+1}\right)}_{R_{c}=x}\\ & = & -\kappa (x-R_{c-1})-\kappa (x-R_{c+1}), \end{array}$$ (7)

where the potential *ϕ* is defined in Eq (2). We take for now a potential well localized at the origin (***μ*** = 0) in Eq (6). Moreover, the pdf at equilibrium is the Boltzmann distribution, conditioned on ***R***_*c*_ = ***x***, that is

$$\begin{array}{c} P\left(R|R_{c}=x\right)=𝓝e^{-\phi (R_{1},...,R_{c-1},x,R_{c+1},..,R_{N})}, \end{array}$$ (8)

where the normalization factor is

$$\begin{array}{ccc} {𝓝}^{-1} & = & \int_{\Omega }..\int_{\Omega }\prod_{i\neq c}P\left(R|R_{c}=x\right). \end{array}$$ (9)

Finally, computing Gaussian integrals (see S1 Text for details) we find that the normalization factor is (for ***μ*** = 0, otherwise we need to replace ***x*** by ***x*** − ***μ***),

$$\frac{1}{N}={\left[\frac{{\left(2\pi \right)}^{N-1}\kappa^{2-N}}{\left(\kappa +\left|c-n\right|k\right)}\right]}^{3/2}\;e^{-\frac{x^{2}(\kappa^{2}+(c\;-\;n\;+\;1)k\kappa )}{2(\kappa \;+\;(c\;-\;n)k)}}.$$ (10)

Substituting Eqs (7)–(10) into Eq (4), we obtain (S1 Text) an explicit inversion formula for the mean velocity of monomer *c*.

**Second inversion formula:**

$$\begin{array}{ccc} \lim_{\Delta t\to 0}E\left\{\frac{R_{c}\left(t+\Delta t\right)-R_{c}\left(t\right)}{\Delta t}|R_{c}\left(t\right)=x\right\} & = & -Dk_{cn}x,\\ k_{cn} & = & \frac{k\kappa }{\kappa +(c-n)k}. \end{array}$$ (11)

Expression 11 is one the key result here: it links the average velocity over empirical trajectories of the observed monomer *c* to a permanent force applied on monomer *n*. The coefficient *k*_*cn*_ depends on the harmonic well strength *k*, the inter-monomer spring constant *κ* and is inversely proportional to the distance ∣*n* − *c*∣ between monomers *n* and *c* along the chain. Furthermore, the steady state variance ***R***_*c*_ = lim_*t* → ∞_ ***R***_*c*_(*t*) of the monomer’s position (see S1 Text) can be related to the dimension d and the coefficient *k*_*cn*_ by

$$\begin{array}{c} \left\langle R_{c}^{2}\right\rangle =\frac{d}{k_{cn}}, \end{array}$$ (12)

when ⟨***R***_*c*_⟩ = 0. Relation 12 is reminiscent of long time asymptotic of classical Ornstein-Uhlenbeck processes. The dynamics of monomer ***R***_*c*_ generated by Brownian simulations is shown in Fig 2b and 2c. In the limit of large *k* (pinned monomer), an analogue of formula 12 was used for analyzing chromatin organization [28] and DNA [34]. Inversion formula 1 assumes the Boltzmann distribution for the single monomer and that the entire polymer has reached equilibrium at the time scale of the simulation or the experiment (from Eq 8). Finally, formula 1 reveals how internal and external polymer forces mix together to influence the monomer velocity. It also shows the explicit decay of the force amplitude with the distance between the observed and forced monomer.

### Locus dynamics for a polymer constricted by two potential wells

We now study the consequences on the motion of a DNA locus of two forces acting on two monomers, located on two opposite sites of the tracked locus. The two monomers *n* and *m* (*n* < *m*) are interacting with two distinct potential wells applied at positions ***μ***_*n*_ and ***μ***_*m*_ (Fig 1a), the total potential energy of the Rouse polymer is

$$\begin{array}{c} U_{\text{ext}}\left(R\right)=\frac{1}{2}k_{n}{\left(R_{n}-\mu_{n}\right)}^{2}+\frac{1}{2}k_{m}{\left(R_{m}-\mu_{m}\right)}^{2}, \end{array}$$ (13)

In that case, the average steady state position of the tagged monomer *c* can be computed exactly and is given by (see S1 Text for details)

$$\left\langle R_{c}\right\rangle =\{\begin{array}{cc} \frac{\mu_{n}k_{n}\left(\kappa +|m-c\right|k_{m})+\mu_{m}k_{m}\left(\kappa +|c-n\right|k_{n})}{k_{n}k_{m}\left|m-n\right|+\left(k_{n}+k_{m}\right)\kappa }, & n<c<m,\\ \frac{k_{n}\mu_{n}\kappa +k_{m}\mu_{m}\left(\kappa +|m-n\right|k_{n})}{k_{n}k_{m}\left|m-n\right|+\kappa \left(k_{n}+k_{m}\right)}, & n<m<c \end{array}$$

and similarly to the previous inversion formula, we can relate the velocity of ***R***_*c*_ to the applied forces, summarized in this new formula

**Third inversion formula**

$$\begin{array}{c} \lim_{\Delta t\to 0}E\left\{\frac{R_{c}\left(t+\Delta t\right)-R_{c}\left(t\right)}{\Delta t}|\tilde{x}\right\}=-Dk_{cnm}\tilde{x}, \end{array}$$ (14)

where $\tilde{x}=x-\langle R_{c}\rangle$ and

$$k_{cnm}=\{\begin{array}{cc} & k_{cn}+k_{cm},\;\text{for}\;n<c<m\\ & \frac{(2\kappa +|m-n|k)k\kappa }{\kappa^{2}+|2c-m-n|k\kappa +|\left(m-n\right)\left(c-m\right)|k^{2}},\;\text{for}\;n<m<c \end{array}$$

where *k*_*cn*_ and *k*_*cm*_ are given by Eq (11) (see S1 Text). For *n* < *m* < *c*, in the limit *m* − *n* ≫ 1, we obtain the limiting formula *k*_*cnm*_ ∼ ∣*c* − *m*∣^−1^ *κ*. Thus, the spring coefficient depends on the distance to the closest anchoring point only. However, when *n* < *c* < *m*, the effective spring coefficient depends on the distance between the two wells. Finally, the variance of the monomer position with respect to its mean position Eq (14) is given by

$$\begin{array}{c} \left\langle {\left(R_{c}-\left\langle R_{c}\right\rangle \right)}^{2}\right\rangle =\frac{d}{k_{cnm}}. \end{array}$$ (15)

The computations are described in the S1 Text. We conclude at this stage that the distance scanned by the tagged monomer is proportional to the distance to the anchoring point (see Fig 1b and 1c). Several interacting forces can certainly be considered, but for a given locus, the two adjacent neighboring interacting monomers are probably enough to characterize the motion, because other forces should be screen by these proximity forces. We shall now extend the inversion formula to other polymer model with a prescribed anomalous exponent.

### Inversion formula for polymer models with a prescribed anomalous exponent

Some refinement of the chromatin dynamics can be accounted for by a class of polymer models (*β*-polymer), generalizing the classical Rouse model. These polymer models account for long-range interactions between monomers, that decay with the distance along the chain [31]. Moreover, the characteristic of this class of model is to specify long-range forces acting on monomers so that a given monomer has a prescribed anomalous exponent [31]. Conversely, once the anomalous exponent is measured, it is then possible to construct a polymer with such given exponent. In that context, deriving an inversion formula for such polymer models is key to relate the velocity of a tagged locus to the external force, where the difficulty is to subtract the long-range internal forces between monomers, associated with the *β*-polymer to the total force and thus to recover the external forces applied to a different monomer than the one observed.

We recall that for a polymer of *N* monomers, the dynamics of monomer *c* is govern by

$$\begin{array}{c} R_{c}=\alpha_{0}^{c}u_{0}+\sum_{p=1}^{N-1}\alpha_{p}^{c}u_{p}, \end{array}$$ (16)

where

$$\alpha_{p}^{c}=\{\begin{array}{cc} \sqrt{\frac{1}{N}},\; & p=0\\ \sqrt{\frac{2}{N}}\cos(\left(c-1/2\right)\frac{p\pi }{N}), & \text{otherwise}. \end{array}$$ (17)

and

$$\begin{array}{c} \frac{du_{p}}{dt}=-D_{p}{\tilde{\kappa }}_{p}u_{p}+\sqrt{2D}\frac{d{\tilde{w}}_{p}}{dt}, \end{array}$$ (18)

where *D*_0_ = *D*/*N* and *D*_*p*_ = *D* (*p* > 0), $\tilde{w_{p}}$ are white noises with mean zero and variance 1, the coefficients are ${\tilde{\kappa }}_{p}=4\kappa \text{sin}{\left(\frac{p\pi }{2N}\right)}^{\beta }$ (2 > *β* > 1). At intermediate time, the cross-correlation function of a locus behaves as

$$\begin{array}{c} \left\langle {\left(R_{c}\left(t_{0}+t\right)-R_{c}\left(t_{0}\right)\right)}^{2}\right\rangle \propto t^{\alpha }, \end{array}$$ (19)

with $\alpha =1-\frac{1}{\beta }$ [31]. When a gradient force (see Eq (6)) acts on monomer ***R***_*n*_ of a *β*-polymer, the expectation of the velocity of monomer *c* (*c* > *n*) is:

**Generalized inversion formula:**

$$\begin{array}{c} \lim_{\Delta t\to 0}E\left\{\frac{R_{c}\left(t+\Delta t\right)-R_{c}\left(t\right)}{\Delta t}|R_{c}\left(t\right)=x\right\}=-Dk_{cn}\left(\beta ,N,l,m\right)x, \end{array}$$ (20)

where ***μ*** = 0 and

$$\begin{array}{c} k_{cn}\left(\beta ,N,l,m\right)=A_{c,c}-\sum_{l,m\neq c}A_{l,c}A_{m,c}{\tilde{C}}_{l,k}^{-1}, \end{array}$$ (21)

where $\tilde{C}$ is a block matrix, the *i*-th block of which is

$$\begin{array}{c} {\tilde{C}}_{j,k}^{i}=A_{j,k}^{i}+k\delta_{i,n}\delta_{j,n}, \end{array}$$ (22)

and [31]

$$\begin{array}{c} A_{j,k}=\sum_{p=0}^{N-1}{\tilde{\kappa }}_{p}\alpha_{p}^{j}\alpha_{p}^{k}. \end{array}$$ (23)

**Fig 3:**
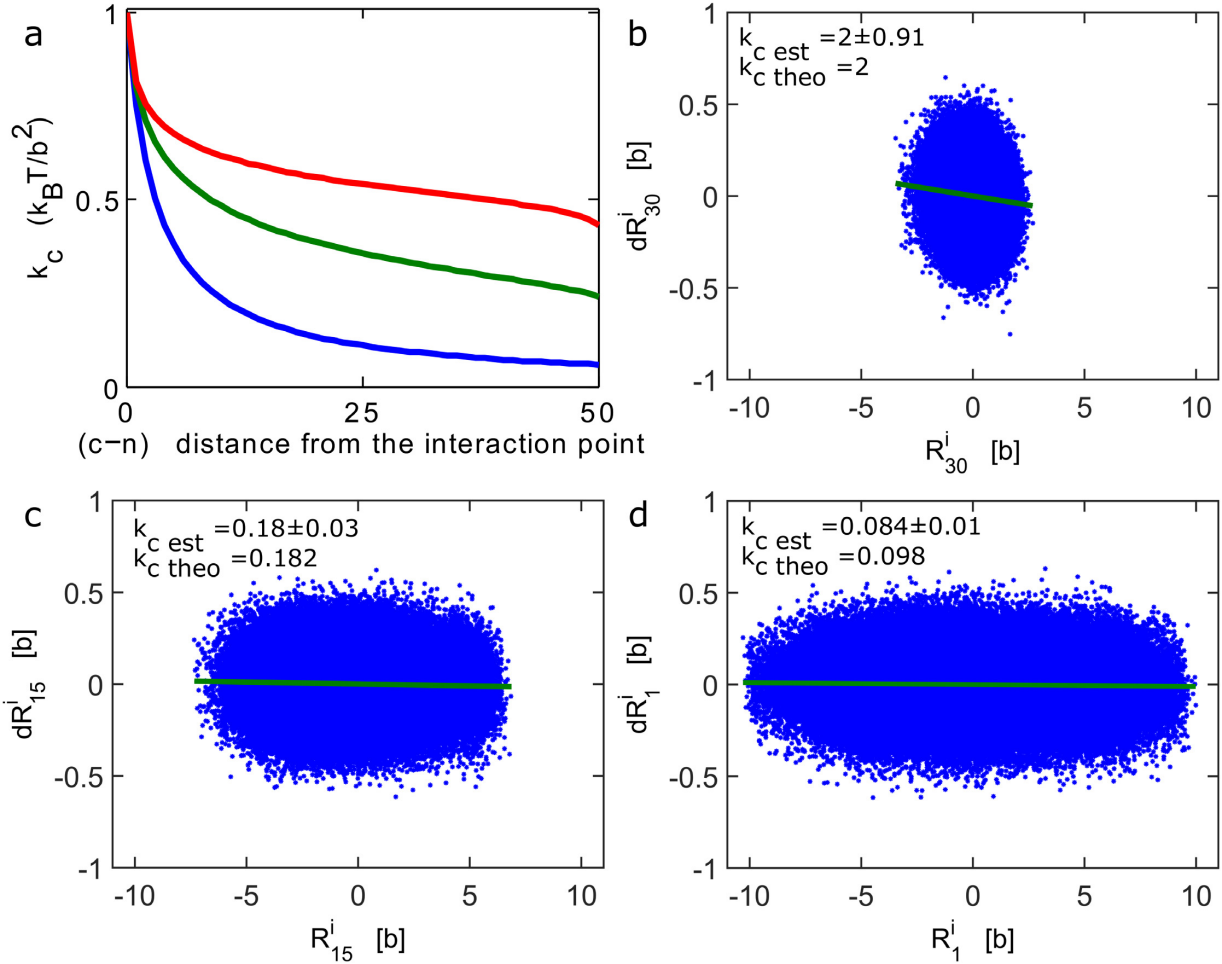
Recovering an external force of an interacting polymer. (**a**)Apparent force acting on a tagged monomer. The apparent spring constant *k*_*c*_ is computed from formula 11 and 20, for a polymer of length *N* = 100, where monomer *n* = 50 interacts with an harmonic potential Eq (6) with *k* = 2*k*_*B*_ *T*/*b* ^2^, while κ = 3*k*_*B*_ *T*/*b* ^2^. The constant *k*_*c*_ is computed for increasing distances |*c* − *n*|, between the observed and the interacting monomers for *β* = 2 (Rouse polymer) (blue), *β* = 1.5 (green) and *β* = 1.2 (red). (**b**-**d**) Brownian simulations of a Rouse polymer (*N* = 30), where the first monomer interacts with a harmonic well at the origin (*k* = 2*k*_*B*_ *T*/*b* ^2^). A scatter plot (blue asterisk) of the steps distribution (*dRi*) against the position for the first monomer (b), middle monomer (c) and end monomer (d). The data clouds are fitted with a linear regression procedure (green line). The apparent spring constant *k*_*c*_ Sim is empirically estimated from simulations using Eq (24) and compared with the theoretical value Eq 11 (*k_cn_* = *kκ*/(*κ*+|*c*-*n*|*k*)). We found *k*_*c* sim_ = 2±91, 0.18±0.03, 0.084±0.01 and *k*_*cn*_ = 2; 0.182; 0.098 respectively, for b, c, d. In the simulations, Δ*t* = 0.01*b* ^2^/*D*.

To conclude, inversion formula Eq 20 for a *β*-polymer is similar to the one derived for a Rouse polymer Eq (11), but the dependency with the parameters is now implicit. Numerical simulations of Eqs 21–23 reveal that the apparent spring constant *k*_*cn*_(*β*, *N*, *l*, *m*) decays slower with the distance ∣*c* − *n*∣ (between the interacting and the observed monomer) for smaller *β* (Fig 3a and 3b). When the chromatin experiences several interactions between distant sites along the chain, the external interactions propagate along the chain.

### Recovering a local force acting on a chromatin locus

In the previous section, we showed how to extract the local interaction between the underlying polymer and the surrounding environment from the trajectories of an observed locus. When the force is applied far away from the tagged locus, it is possible to recover the strength of the force and the distance where it was applied from the statistics of trajectories. The three inversion formulas we derived above can be used for different polymer models. In this section, we apply these formulas to extract parameter from numerical simulations and then we present a computational method to recover forces (chromatin interactions) from trajectories of the MAT-locus imaged in living yeast cells.

#### Empirical estimators to extract from Single particle trajectories, forces acting on a single locus

To extract the empirical effective spring coefficient *k*_*c*_ from a trajectory given by *R*_*c*_(*h*Δ*t*) (*h* = 1..*N*_*p*_), where *N*_*p*_ is the number of points, we start by computing the differential quotient in Eq 11. This first step allows extracting the mean position of the locus. Once the steady state is reached, the time average of the locus position is computed from

$$\begin{array}{c} \left\langle R_{c}\right\rangle \approx \frac{1}{N_{p}}\sum_{h=1}^{N_{p}}R_{c}\left(h\Delta t\right). \end{array}$$

When the polymer interacts with a single interacting potential, the average position **⟨** *R*_*c*_⟩ estimates the location where the force is applied. An upper bound for the number of points *N*_*p*_ is of order (*τ*_*r*_/Δ*t*), where $\tau_{r}=\frac{|c-n{|}^{\beta }}{D\kappa \pi^{\beta }}$ is the relaxation time for a portion of the chain between *c* and *n* of a *β*-polymer [31].

In the next step, we assume that the diffusion coefficient *D* has been estimated, which can be done using second moment estimators [29]. We also consider that the inter-monomer spring constant *κ* is known, which reflects an intrinsic property of the chromatin. To estimate the force from the constant *k*_*cn*_ in Eq 11, we use the linearity of the force with respect to the position of the locus (see Eq 6). The step size (***R***(Δ*t*(*h* + 1))−***R***(Δ*th*)) is thus proportional to the locus position (***R***(Δ*t*(*h* + 1))−⟨***R***_*c*_⟩) (Fig 3b–3d). In the isotropic case, the apparent force constant *k*_*cn*_ acting on monomer *c* is computed from the trajectories of ***R***_*c*_(*t*)

$$\begin{array}{c} k_{c}\approx \frac{1}{d(N_{p}-1)}\sum_{i=1}^{d}\sum_{h=1}^{N_{p}-1}\frac{R_{c}^{i}\left(\left(h+1\right)\Delta t\right)-R_{c}^{i}\left(h\Delta t\right)}{D\Delta t(R_{c}^{i}\left(h\Delta t\right)-\left\langle R_{c}^{i}\right\rangle )}, \end{array}$$ (24)

(*d* is the dimension and *N*_*p*_ is the number of points). To demonstrate the efficiency of inversion formula 1, we ran stochastic simulations of a Rouse polymer and applied the procedure described above with formula 26 to extract from trajectories the coefficient *k*_*cn*_ (Fig 3b–3d). A potential well is applied on the first monomer *n* = 1, and we present three cases where the tagged monomer is the first (*c* = 1), the middle (*c* = *N*/2) or the last one (*c* = *N*). Using a linear regression, we recover the theoretical apparent force constant *k*_*cn*_ in formula (6) from stochastic simulations. Once the parameter *k*_*cn*_ is computed, we are left with two unknown parameters: the spring force *k* and the distance ∣*c* − *n*∣. For a strong anchoring (*k* ≫ *κ*), we can approximate *k*_*c*_ ≈ *κ*∣*c* − *n*∣^−1^. In that case, the empirical effective spring constant can be used to estimate the distance to the interacting monomer.

For a long enough sampled trajectory and a force derived from a stationary potential well, the effective spring coefficient can be recovered directly either from the empirical estimator Eq (24) or by using the reciprocal of the variance Eq (12). However, trajectories are often measured with a small sampling time Δ*t* allowing probing the fine behavior of the chromatin and recovering accurately the diffusion coefficient. The total length of a trajectory is however limited by photo bleaching effects [35]. Thus, the length of a trajectory may be shorter than the equilibration time scale, and thus acquired before equilibrium is reached. In that case, formula Eq (24) can still be applied to recover the parameter *k*_*c*_, while formula Eq 12, which implies equilibrium, cannot be used. The standard error of the mean position is $\sigma /\sqrt{N_{p}}$ (where by definition *σ* ^2^ = ⟨*R*_*c*_ − ⟨*R*_*c*_⟩)^2^⟩) and the standard error of the variance is $\sigma^{2}\sqrt{\frac{2}{N_{p}-1}}$ [36], thus a good estimate of the mean (position) requires less points than for computing the variance.

#### Recovering forces from the auto-correlation function

How is the force applied to a polymer reflected in the auto-correlation function *C*(*c*, *t*_1_, *t*_2_) = ⟨[***R***_*c*_(*t*_1_)−⟨***R***_*c*_(*t*_1_)⟩][***R***_*c*_(*t*_2_)−⟨***R***_*c*_(*t*_2_)⟩]⟩ of the tagged monomer *c*? We shall demonstrate here that the auto-correlation function can be used to recover the spring constant *k*. Indeed, by decomposing the external potential Eq (6) on the basis ***u***_*p*_, that diagonalizes the Rouse potential Eq (17), we get

$$U_{ext}\left(R_{n}\right)=\frac{1}{2}k\;{\left(\mu -\overset{N-1}{\underset{p=0}{\sum }}\;\alpha_{p}^{n}u_{p}\right)}^{2}$$ (25)

and the Rouse Eq (18) for the polymer are

$$\begin{array}{c} \frac{du_{p}}{dt}=D(k\alpha_{p}^{n}\mu -\left({\left(\alpha_{p}^{n}\right)}^{2}k+{\tilde{\kappa }}_{p}\right)u_{p})-Dk\alpha_{p}^{n}\sum_{q=0,q\neq p}^{N-1}\alpha_{q}^{n}u_{q}x+\sqrt{2D}\frac{d{\tilde{w}}_{p}}{dt}. \end{array}$$ (26)

for *p* = 0..*N*−1. The force applied on monomer *n* couples the modes dynamics (there are non-diagonal terms). However, when the strength of the coupling term is relatively weak ${\left(\alpha^{n_{p}}\right)}^{2}k\ll {\tilde{\kappa }}_{p}$, we can neglect the coupling. This will be the case for higher modes given that *k* < *κ* and *N* large. Thus the expansion of the auto-correlation function is

$$\begin{array}{c} C\left(c,t_{1},t_{2}\right)=\frac{d}{k}e^{-D{\left(\alpha_{0}^{n}\right)}^{2}k\left(t_{2}-t_{1}\right)}+\sum_{p=1}^{N-1}\frac{d{\left(\alpha_{p}^{c}\right)}^{2}}{{\left(\alpha_{p}^{n}\right)}^{2}k+{\tilde{\kappa }}_{p}}e^{-D\left({\left(\alpha_{p}^{n}\right)}^{2}k+{\tilde{\kappa }}_{p}\right)\left(t_{2}-t_{1}\right)}. \end{array}$$ (27)

Thus the auto-correlation function decays exponentially and the exponent of the dominant term is proportional to $D{\left(\alpha_{0}^{n}\right)}^{2}k$. Thus when the diffusion coefficient *D* is known, it is possible to extract the spring constant *k*.

#### Extracting forces from live cell imaging in yeast

**Fig 4:**
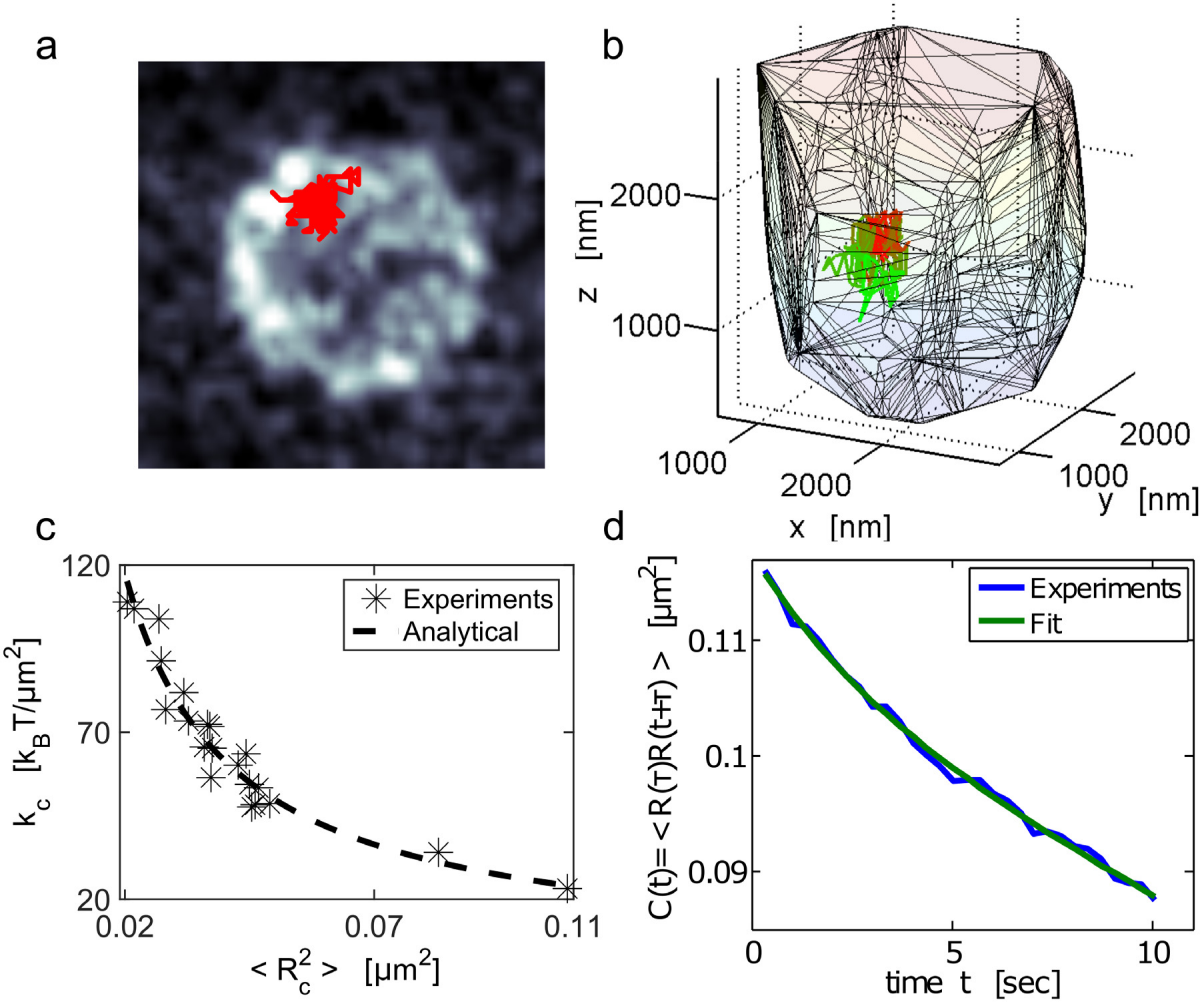
Single locus dynamics and mean applied force on the Yeast chromatin. (**a**) Trajectory of the chromatin MAT-locus located on chromosome III in the yeast SS. The locus trajectory (red) inside the nucleus is projected on the XY plane. The nuclear membrane (gray scale) was stained with the nup49-mCherry fusion protein. The time resolution is Δ*t* = 0.33 seconds during an acquisition time of approximately 100 seconds. (**b**) Three-dimensional trajectories: the color codes for time propagation. Initially (*t* = 0) the trajectory is red and gradually becomes green (*t* = 100sec). The convex hull is the nuclear envelope reconstruction. (**c**) Scatter plot of the effective spring coefficient *k*_*c*_ and the variance ($R_{c}^{2}$) of the locus trajectory estimated in two-dimensions, extracted for 21 cells. The constant *k*_*c*_ is estimated using formula Eq (24), fitted to a power law, $k_{c}=\frac{a}{{\langle R_{c}^{2}\rangle }^{b}}$, with *a* = 3.03 ± 1.05 *k*_*B*_ *T* and *b* = 0.94 ± 0.1. (**d**) Auto-correlation function computed using formula Eq (28) for the trajectory shown in a. The fit uses the sum of two exponentials: $C(t)=a_{1}e^{-t/\tau_{1}}+a_{2}e^{-t/\tau_{2}}$, with *τ*_1_ = 45.7 ± 0.005*s* and *τ*_2_ = 2.4 ± 0.35 s, *a*_1_ = 109 ± 5 × 10^−3^ *μm* ^2^, *a*_2_ = 8.38 ± 4.94 × 10^−3^ *μm* ^2^.

We now apply the present analysis to the dynamics of a chromatin locus. We monitored the time fluctuations of the chromatin fiber by following a GFP tagged DNA locus in the yeast *S. cerevisiae* (see materials and methods and [24]). We followed the MAT-locus (Fig 4a and 4b) for 100sec with a time resolution of Δ*t* = 0.33sec and found that the trajectories were exploring a small region of the nuclear volume. The trajectory shown in Fig 4a was contained in a ball of radius 221nm (the nucleus is approximately a ball of the radius 1.5*μ*m). Thus the locus is restricted to a small region of the nucleus. To extract the possible forces constraining this motion, we analyzed independently the trajectories of the MAT-locus in several cells. As cells are observed in the G1 phase, this analysis assumes that interactions on the chromatin do not change transiently, but rather have reached steady state, compared to the time scale of few minutes of the recording.

Within the hypothesis that the chromatin was interacting locally with other nuclear elements, we extracted the overall force resulting from these interactions by applying formula Eq (24) to estimate the effective force constant *k*_*c*_ from the sampled trajectories. The trajectories are acquired in three dimensions. However, due to the precision difference in the XY plane (65nm×65nm) compare to the *z* axis (300nm), we only used the *x*− and *y*− projections to evaluate the constant *k*_*c*_.

Applying the extraction procedure to 21 cells, we found a large heterogeneity between cells for the values of *k*_*c*_ (Fig 4c), with a mean of ⟨*k*_*c*_⟩ = 67 ± 22*k*_*B*_ *T*/*μ*m^2^. This heterogeneity suggests that in different cells the locus interacts differently with various nuclear elements. To verify that the motion of the chromatin locus is indeed impacted by external interactions, we plotted in Fig 4c, the force constant *k*_*c*_ for each cell with respect to the locus position averaged over trajectories, (empirically estimated by $\langle R_{c}^{2}\rangle =\frac{1}{T}\overset{N_{p}{}_{h=1}}{\sum }{\left(R_{c}(h\Delta t)-\langle R_{c}\rangle \right)}^{2}$). The distribution of points confirms the prediction of the power law relation 17 between *k*_*c*_ and $\left\langle R_{c}^{2}\right\rangle$, given by $k_{c}=\frac{3.0}{{\langle R_{c}^{2}\rangle }^{0.94}}$. This relation was predicted by formula Eq (12), although the expected value for the coefficient is *d* = 2 and not 3. This relation should hold for long trajectories so that the equilibrium distribution is sufficiently sampled. This condition may not hold in general, but the power law decay suggests that the origin of the localization is due to interactions.

To test that the polymer model we are using, gives a self-consistent framework for interpreting the MAT-locus dynamics, we computed the auto-correlation function for the MAT-locus trajectory [33],

$$\begin{array}{c} C\left(t\right)=\frac{1}{N_{p}-t}\sum_{i=1}^{d}\sum_{k=1}^{N_{p}-t}R_{c}^{i}\left(k\Delta t\right)R_{c}^{i}\left(\left(k+1\right)\Delta t\right), \end{array}$$ (28)

in Fig 4d. Fractional Brownian motion has been previously used to model the dynamics of chromatin loci [1, 18, 37]. For fractional Brownian motion, the auto-correlation function *C*(*t*) decays as a sum of power laws [38]. Thus we first fitted *C*(*t*) with a power law, but could not obtained a satisfactory approximation, suggesting that the description of the locus motion as a fractional Brownian motion alone is not sufficient. However, we obtain a good fitting of the function *C*(*t*) by a sum of two exponentials

$$\begin{array}{c} C\left(t\right)\approx a_{1}e^{-t/\tau_{1}}+a_{2}e^{-t/\tau_{2}}, \end{array}$$

with *τ*_1_ = 45.7 ± 0.005sec,*τ*_2_ = 2.4 ± 0.35sec and *a*_1_ = 109 ± 5 × 10^−3^ *μ*m^2^, *a*_2_ = 8.38 ± 4.94 × 10^−3^ *μ*m^2^. This fit suggests that the auto-correlation function for the locus position is well described by a sum of two exponentials, as predicted by formula Eq (27) derived for general polymer model.

We conclude that polymer models, such as Rouse or *β*− polymer account for the dynamics of a chromatin locus. In that context, it was possible to extract from SPTs, characteristics of the DNA locus, its dynamics, external forces and some properties of the polymer model. At this stage, we cannot determine the nature and physical origin of the anchoring forces. Forces may occur at the centromere, which is anchored to the nuclear membrane in yeast through interaction with the spindle pole body and/or at the telomeres, which are interacting with the nuclear membrane through several pathways [39]. The large variability of the locus position suggests that the extracted forces can happen at sub-telomere regions or with other chromosomes. Future investigations are needed to clarify the nature of these measured forces.

## Discussion

We have shown here how to extract from single locus trajectories, chromatin tethering mediated by interactions with its surrounding environment. The presented method allows recovering an external force applied on chromatin although this one occurs far away from the observed locus. We note that this analysis is valid, although the recorded trajectories are possibly shorter than the relaxation time of the anchored chromatin. However, it is not yet possible to discern the forces from a locus positioned between two different interacting potential wells from the one generated by a single force located far away from the observed locus. In the complex nuclear environment, interactions of different strength can be randomly and transiently distributed along the chromatin. However, the resulting force on a single locus should mostly be generated by the sum of the two nearest interacting forces (derived from two stable potential wells). The distribution of the spring values *k*_*c*_ shown in Fig 4c can be attributed to different interaction strength (*k*—Eq 6) or to the distances between the observed locus and the nearest interacting wells Eq (15). Other traps beyond the two nearest ones should certainly have an additional but lower contribution that needs to be estimated.

A refined description of interactions on the chromatin would require monitoring simultaneously several loci. The present approach is also applicable for higher order organized polymer, modeled by *β*-polymers and we extracted here *in vivo* interactions of the chromatin with other nuclear element that were reflected in the motion of the MAT-locus. These interactions are responsible for constraining the locus in a small fraction of the nucleus.

The motion of the chromatin is driven by both thermal fluctuations and by active ATP-dependent forces [25]. While our modeling is relevant to extract an interaction that does not change during the time acquisition of the trajectory, the spring constant *k*_*c*_ that would be extracted during an active chromatin motion could be differentiated from the thermal one by projecting the dynamics perpendicular to the direction of motion. Finally, the present approach could also be used to study how chromatin modifications occurring during gene transcription or double stranded DNA repair affect the dynamics of a given locus.

## Materials and Methods

### Experimental procedure: Yeast and growth conditions

Yeast strains used in this study are all derivatives of the JKM179 strain [11] which is MAT*α* ade1 leu2-3, leu2-112 lys5 trp1::HisG ura3-52. The strain was obtained through insertion of both a Lac operator array (256 lacOp repeats), a Nup49-mCherry fusion and a non-tetramerizing lac repressor-GFP fusion under the HIS3 promoter into JKM179. To serve as a static reference point in the nucleus, the Spc42 protein was fused to yEGFP. All insertions or deletions were verified by PCR and phenotypic assays.

#### Movies analysis

Microscopy Images were captured with a ×100 magnification oil-immersion objective (1.46 numerical aperture) on a Leica DMI 6000B microscope (Leica Microsystems) equipped with a piezoelectric translator (PIFOC, Physik Instrumente), a ORCA-Flash 4.0 camera (Hamamatsu) an illumination system with leds (Lumencore) and rapid imaging software (Metamorph). Wavelengths of the leds used are 475nm (for GFP, 205mW), and/or 575nm (for mCherry, 300MW). Two-minute movies with a stack of 10 optical slices separated by 300nm every 338ms. Each slice was exposed for 30ms for a total of 338ms per stack. All microscopy was done in a temperature-controlled environment set to 25°C. The raw images were deconvolved using the Autoquant software. The movies were then tracking using ImageJ [40] with the Mosaic macro [41] to produce 3D+t trajectories. Further processing and analysis of the movies was done using Matlab.

#### Brownian simulations

To simulate the dynamics of the polymer, we used the Euler’s method to discretize the equations into

$$\begin{array}{c} dR_{n}=\nabla_{n}\phi \left(R\right)dt+\sqrt{2D}dw, \end{array}$$ (29)

where *ϕ*(***R***) is given by Eq (2), *D* is the diffusion coefficient and ***w*** are the three dimensional white Gaussian noise, with mean zero and variance 1.

## Supporting Information

S1 Text This Supplementary information contains the detail of the computations and analysis to extract the strength of a potential well for a Rouse polymer. (PDF)
