# Supplementary material for: Analysis of Single Locus Trajectories for Extracting In Vivo Chromatin Tethering Interactions
Source: PLoS Comput Biol. 2015 Aug 28;11(8):e1004433. doi: 10.1371/journal.pcbi.1004433 (PMC4552938; doi:10.1371/journal.pcbi.1004433)
Supplement: S1 Text — (PDF) [file pcbi.1004433.s001.pdf]

Supplementary Information: Analysis of single locus trajectories for extracting in vivo chromatin tethering interactions

Amitai Assaf[1], Toulouze Mathias[2], Dubrana Karine[2], Holcman David[3,4,*],
1 Institute for Medical Engineering & Science, Massachusetts Institute of Technology, Cambridge, MA, USA.
2 Laboratory of genetic instability and nuclear organization, CEA, 92265 Fontenay-aux-Roses, France.
3 IBENS, Ecole Normale Supérieure, 46 rue d'Ulm 75005 Paris, France.
4 Mathematical Institute University of Oxford, Oxford OX2 6GG UK.

Supplementary Information

In this Supplementary Information, we detail the analysis, calculations and results presented in the main text.

Extracting the strength of a potential well for a Rouse polymer

A Rouse polymer which has one monomer interacting with a infinite potential well, can be described by the energy potential

$$\Phi(\boldsymbol{R}) = \frac{k}{2}\boldsymbol{R}_n^2 + \frac{\kappa}{2}\sum_{i=1}^{N-1}(\boldsymbol{R}_{i+1} - \boldsymbol{R}_i)^2. \tag{1}$$

where $\boldsymbol{R} = (\boldsymbol{R}_1, \boldsymbol{R}_2, ..., \boldsymbol{R}_N)$ is the collection of the monomers, connected by a spring constant $\kappa = dk_BT/b^2$, b is the standard-deviation of the distance between adjacent monomers [21], k_B the Boltzmann coefficient, T the temperature and d the dimensionality (dim 2 or 3), k is the strength of the external harmonic well acting on monomer n, located at the origin.
To extract the strength of the potential well applied on monomer n from the measured velocity of locus c $(n < c)$, we will compute

$$\lim_{\Delta t \to 0} \mathbb{E}\{\frac{\boldsymbol{R}_c(t+\Delta t) - \boldsymbol{R}_c(t)}{\Delta t}|\boldsymbol{R}_c = \boldsymbol{x}\} = -D\int_\Omega d\boldsymbol{R}_1..\int_\Omega d\boldsymbol{R}_N(\nabla_{\boldsymbol{R}_c}\Phi)P(\boldsymbol{R}|\boldsymbol{R}_c = \boldsymbol{x}). \tag{2}$$

The force acting on monomer c, when its position is $\boldsymbol{x}$ is given by

$$\boldsymbol{F}^{c}_{\boldsymbol{R}_c=\boldsymbol{x}} = -\nabla_{\boldsymbol{R}_c}\Phi(\boldsymbol{R}_{c-1},\boldsymbol{R}_c,\boldsymbol{R}_{c+1})_{\boldsymbol{R}_c=\boldsymbol{x}} = -\kappa\left(\boldsymbol{x}-\boldsymbol{R}_{c-1}\right)-\kappa\left(\boldsymbol{x}-\boldsymbol{R}_{c+1}\right). \quad (3)$$

The equilibrium probability distribution function is the Boltzmann distribution, conditioned to $\boldsymbol{R}_c=\boldsymbol{x}$:

$$P(\boldsymbol{R}|\boldsymbol{R}_c=\boldsymbol{x}) = \mathcal{N}e^{-\Phi(\boldsymbol{R}_1,\ldots,\boldsymbol{R}_{c-1},\boldsymbol{x},\boldsymbol{R}_{c+1},..,\boldsymbol{R}_N)}, \quad (4)$$

with the normalization factor

$$\begin{aligned}
\mathcal{N}^{-1} &= \int_\Omega .. \int_\Omega d\boldsymbol{R}_1..d\boldsymbol{R}_{c-1}d\boldsymbol{R}_{c+1}..d\boldsymbol{R}_N P(\boldsymbol{R}|\boldsymbol{R}_c=\boldsymbol{x}) \\
&= \int \exp\left[-\kappa\boldsymbol{x}^2\right]\exp\left[-\frac{1}{2}\sum_{p,q=1;p,q\neq c}^{N} A_{p,q}R_pR_q + \sum_{p=1;p\neq c}^{N} B_pR_p\right] d\boldsymbol{R}_1..d\boldsymbol{R}_{c-1}...d\boldsymbol{R}_N \\
&= \left[\frac{(2\pi)^{N-1}}{\mathrm{detA}}\right]^{d/2} e^{-\kappa\boldsymbol{x}^2}e^{\frac{1}{2}\boldsymbol{B}^TA^{-1}\boldsymbol{B}}. \quad (5)
\end{aligned}$$

The matrix A is a matrix that can be decomposed into d blocks A^i, each of size $(N-1)\times(N-1)$. A^i is also a block matrix of

$$A^i = \begin{pmatrix} A^i_1 & 0 \\ 0 & A^i_2 \end{pmatrix} \quad (6)$$

with

$$A^i_1 = \begin{pmatrix}
\kappa & -\kappa & 0 & \cdots & 0 & 0 \\
-\kappa & 2\kappa & -\kappa & \cdots & 0 & 0 \\
\vdots & \vdots & \vdots & \ddots & \vdots & \vdots \\
\vdots & 0 & -\kappa & 2\kappa+k & -\kappa & \vdots \\
\vdots & \vdots & \vdots & \ddots & \vdots & \vdots \\
\vdots & 0 & 0 & -\kappa & 2\kappa & -\kappa \\
\vdots & 0 & 0 & 0 & -\kappa & 2\kappa
\end{pmatrix}, \quad (7)$$

which is of size $(c-1)\times(c-1)$ and

$$A^i_2 = \begin{pmatrix}
2\kappa & -\kappa & 0 & \cdots & 0 & 0 \\
-\kappa & 2\kappa & -\kappa & \cdots & 0 & 0 \\
\vdots & \vdots & \vdots & \ddots & \vdots & \vdots \\
\vdots & 0 & 0 & -\kappa & 2\kappa & -\kappa \\
\vdots & 0 & 0 & 0 & -\kappa & \kappa
\end{pmatrix}, \quad (8)$$

which is of size $(N-c) \times (N-c)$. The vector $\boldsymbol{B}$ is composed of d blocks, each given by

$$B^i = \begin{pmatrix} 0 \\ \vdots \\ \kappa x^i \\ \kappa x^i \\ 0 \\ \vdots \end{pmatrix}. \tag{9}$$

Since A^i is a tridiagonal matrix, we compute the determinant and its inverse using an algorithm proposed in [34]: first, the determinant of the matrix A^i is found by solving the recurrence relation

$$\begin{aligned} &\theta_l = b_l\theta_{l-1} - a_l c_{l-1}\theta_{l-2} \;\; l \geq 1, \\ &\theta_{-1} = 0, \\ &\theta_0 = 1, \end{aligned} \tag{10}$$

where b_j are the elements on the diagonal ($j = 1..N$), a_j are the elements bellow the diagonal ($j = 2..N$) and c_j are the elements above the diagonal ($j = 1..N-1$). For $l < n$ the recurrence relation is

$$\begin{aligned} &\theta_l = 2\kappa\theta_{l-1} - \kappa^2\theta_{l-2} \;\; \text{for } n > l \geq 2, \\ &\theta_0 = 1, \\ &\theta_1 = \kappa. \end{aligned} \tag{11}$$

The characteristic polynomial of the relation is

$$P(t) = t^2 - 2\kappa t + \kappa^2, \tag{12}$$

which has one double root

$$r = \kappa. \tag{13}$$

Thus, the solution of the recurrence relation (11) is

$$\theta_l = k_1\kappa^l + k_2 l\kappa^l. \tag{14}$$

Substituting the initial conditions (eq.11), we find

$$\theta_l = \kappa^l \;\; \text{for } l < n. \tag{15}$$

We next find the series solution at position n:

$$\theta_n = (2\kappa + k)\theta_{n-1} - \kappa^2\theta_{n-2} = \kappa^n + k\kappa^{n-1}, \tag{16}$$

while

$$\theta_{n+1} = 2\kappa\theta_n - \kappa^2\theta_{n-1} = \kappa^{n+1} + 2k\kappa^n. \tag{17}$$

We solve again the recurrence relation

$$\begin{aligned}
&\theta_l = 2\kappa\theta_{l-1} - \kappa^2\theta_{l-2} \ \text{ for } l > n+1, \\
&\theta_n = \kappa^n + k\kappa^{n-1}, \\
&\theta_{n+1} = \kappa^{n+1} + 2k\kappa^n.
\end{aligned} \tag{18}$$

Eq.(18) has the same characteristic polynomial (eq.12), thus

$$\theta_l = k_1\kappa^l + k_2 l\kappa^l \tag{19}$$

and with the new initial conditions (eq.18), we find

$$\theta_l = \kappa^{n-1}\left(\kappa^{l-n+1} + (l-n+1)k\kappa^{l-n}\right), \quad \text{for } n \le l \le c-1. \tag{20}$$

Thus, since $\det A_1^i = \theta_{c-1}$ [34]:

$$\det A_1^i = \kappa^{n-1}\left(\kappa^{c-n-1+1} + (c-1-n+1)k\kappa^{c-1-n}\right) = \left(\kappa^{c-1} + (c-n)k\kappa^{c-2}\right) \tag{21}$$

and using eq.(15) we find

$$\det A_2^i = \kappa^{N-c}. \tag{22}$$

The term in the exponential in eq.(5) is given by

$$(B^i)^T[A^i]^{-1}B^i = (\kappa x^i)^2\left((A_1^i)^{-1}_{c-1,c-1} + (A_2^i)^{-1}_{1,1}\right). \tag{23}$$

Since A_1^i is a tridiagonal matrix [34]

$$(A_1^i)^{-1}_{c-1,c-1} = \frac{\theta_{c-2}}{\theta_{c-1}} = \frac{\kappa^{-1}\left(\kappa^{c-1} + (c-n-1)k\kappa^{c-2}\right)}{\left(\kappa^{c-1} + (c-n)k\kappa^{c-2}\right)} = \frac{\kappa + (c-n-1)k}{\kappa\left(\kappa + (c-n)k\right)}, \tag{24}$$

while [34]

$$(A_2^i)^{-1}_{1,1} = \frac{1}{\kappa}. \tag{25}$$

Substituting (24) and (25) into (23) we find

$$(B^i)^T[A^i]^{-1}B^i = (\kappa x^i)^2\left(\frac{\kappa + (c-n-1)k}{\kappa\left(\kappa + (c-n)k\right)} + \frac{1}{\kappa}\right) = (\kappa x^i)^2\left(\frac{2}{\kappa} - \frac{k}{\kappa\left(\kappa + (c-n)k\right)}\right). \tag{26}$$

Substituting (26) into (5) we find

$$\begin{aligned}
\mathcal{N}^{-1} &= \left[\frac{(2\pi)^{N-1}}{\left(\kappa + (c-n)k\right)\kappa^{N-2}}\right]^{3/2} e^{-\kappa\boldsymbol{x}^2} e^{\frac{1}{2}\boldsymbol{B}^T A^{-1}\boldsymbol{B}} \\
&= \left[\frac{(2\pi)^{N-1}}{\left(\kappa + (c-n)k\right)\kappa^{N-2}}\right]^{3/2} e^{-\frac{1}{2}\kappa\boldsymbol{x}^2} e^{-\frac{\boldsymbol{x}^2 k\kappa}{2\left(\kappa + (c-n)k\right)}}.
\end{aligned} \tag{27}$$

We can now we calculate the conditional expectation of the measured velocity of monomer c (eq.2).

$$\begin{aligned}
&\lim_{\Delta t \to 0} \mathbb{E}\{\frac{\boldsymbol{R}_c(t+\Delta t)-\boldsymbol{R}_c(t)}{\Delta t}|\boldsymbol{R}_c=\boldsymbol{x}\} = \\
&-\mathcal{N}De^{-\kappa \boldsymbol{x}^2}\int \left(\kappa\left(\boldsymbol{x}-\boldsymbol{R}_{c-1}\right)-\kappa\left(\boldsymbol{R}_{c+1}-\boldsymbol{x}\right)\right)e^{-\frac{1}{2}\sum_{i,p,q}A^i_{p,q}R_pR_q+\sum_{i,p}B^i_pR^i_p}\prod_{i\neq c}^{N} d\boldsymbol{R}_i = \\
= \quad & -\mathcal{N}D\left[\frac{(2\pi)^{N-1}}{\det\mathrm{A}}\right]^{3/2}e^{-\kappa \boldsymbol{x}^2}e^{\frac{1}{2}B^TA^{-1}B}\kappa\left(2\boldsymbol{x}-\left(B_{c-1,c-1}(A^i_1)^{-1}_{c-1,c-1}+B_{c,c}(A^i_2)^{-1}_{1,1}\right)\right) = \\
&= -D\kappa\left(2\boldsymbol{x}-\kappa\boldsymbol{x}\left(\frac{2}{\kappa}-\frac{k}{\kappa\left(\kappa+(c-n)k\right)}\right)\right) = -D\frac{k\kappa\boldsymbol{x}}{\kappa+(c-n)k}. \qquad (28)
\end{aligned}$$

Finally

$$\lim_{\Delta t \to 0} \mathbb{E}\{\frac{\boldsymbol{R}_c(t+\Delta t)-\boldsymbol{R}_c(t)}{\Delta t}|\boldsymbol{R}_c=\boldsymbol{x}\} = -\boldsymbol{x}Dk_{cn}, \qquad (29)$$

where

$$k_{cn} = \frac{k\kappa}{\kappa+(c-n)k}. \qquad (30)$$

For a Rouse polymer the force depends only on the distance along the chain $|c-n|$. Interestingly, the restoring force decays inversely proportional to the distance along the chain.

0.1 The variance of monomer c position

We now compute the variance of a monomer position $\langle \boldsymbol{R}_c^2\rangle$ with respect to its average position (mean zero)

$$\begin{aligned}
\langle \boldsymbol{R}_c^2\rangle \quad &= \quad \int..\int \boldsymbol{R}_c^2P(\boldsymbol{R})\prod_{i=1}^{N}d\boldsymbol{R}_i = \mathcal{N}\int \boldsymbol{R}_c^2e^{-\Phi(\boldsymbol{R})}\prod_{i=1}^{N}d\boldsymbol{R}_i \\
&= \quad \mathcal{N}\int \boldsymbol{R}_c^2\exp\left[-\frac{1}{2}\sum_{p,q=1;p,q\neq c}^{N}G_{p,q}R_pR_q\right]\prod_{i=1}^{N}d\boldsymbol{R}_i, \qquad (31)
\end{aligned}$$

where the potential Φ is given by (1) and G is a matrix composed of d blocks (G^i), each of size $N \times N$:

$$G^i = \begin{pmatrix} \kappa & -\kappa & 0 & \cdots & 0 & 0 \\ -\kappa & 2\kappa & -\kappa & \cdots & 0 & 0 \\ \vdots & \vdots & \vdots & \ddots & \vdots & \vdots \\ \vdots & 0 & -\kappa & 2\kappa + k & -\kappa & \vdots \\ \vdots & \vdots & \vdots & \ddots & \vdots & \vdots \\ \vdots & 0 & 0 & -\kappa & 2\kappa & -\kappa \\ \vdots & 0 & 0 & 0 & -\kappa & \kappa \end{pmatrix}. \tag{32}$$

Thus, rewriting eq.(31)

$$\begin{aligned} \langle \boldsymbol{R}_c^2 \rangle &= \mathcal{N} \int \boldsymbol{R}_c^2 \exp\left[-\frac{1}{2} \sum_{p,q=1;p,q\neq c}^{N} G_{p,q} R_p R_q \right] \prod_{i=1}^{N} d\boldsymbol{R}_i \\ &= \mathcal{N} \left[\frac{(2\pi)^{d(N-1)}}{\det G} \right]^{1/2} \sum_i (G^i)^{-1}_{cc} = \sum_i (G^i)^{-1}_{cc}, \end{aligned} \tag{33}$$

where [34]

$$(G^i)^{-1}_{cc} = \frac{\theta_{c-1}\phi_{c+1}}{\det G} \tag{34}$$

and

$$\begin{aligned} &\phi_l = b_l \theta_{l+1} - a_{l+1} c_l \theta_{l+2} \quad \text{for } n < l \leq N, \\ &\phi_{N+1} = 1, \\ &\phi_{N+2} = 0. \end{aligned} \tag{35}$$

Since $c > n$, solving the recurrence relation 35, we find

$$\phi_l = \kappa^{N-l+1} \quad \text{for} \quad \text{for } n < l \leq N, \tag{36}$$

Thus, using the value θ_{c-1} (eq.20)

$$\begin{aligned} \phi_{c+1} &= \kappa^{N-c} \\ \theta_{c-1} &= \kappa^{n-1} \left(\kappa^{c-n} + (c-n) k \kappa^{c-n-1} \right), \end{aligned} \tag{37}$$

while

$$\theta_{N-1} = \kappa^{n-1} \left(\kappa^{N-n} + (N-n) k \kappa^{N-n-1} \right) \tag{38}$$

Finally, the determinant is given by

$$\det G = \kappa\theta_{N-1} - \kappa^2\theta_{N-2} = k\kappa^{N-1} \tag{39}$$

and the inverse element

$$(G^i)^{-1}_{cc} = \frac{\theta_{c-1}\phi_{c+1}}{\det G} = \frac{\kappa + (c-n)k}{k\kappa} \tag{40}$$

Finally, substituting (40) into eq.(33), we find the variance for the position to be

$$\langle \boldsymbol{R}_c^2 \rangle = \sum_i \frac{\kappa + (c-n)k}{k\kappa} = \frac{d}{k_{cn}}, \tag{41}$$

where we use the definition 30 for k_{cn}.

0.2 Extracting the strength of a potential well for a β-polymer

We now derive an expression for the measured velocity of a monomer c when monomer n further away along the chain interacts with an harmonic potential well for the β-polymer model [24]. We recall that for the β-model, the polymer potential is given

$$\tilde{\phi}(\boldsymbol{R}_1, ..., \boldsymbol{R}_N) = \frac{1}{2}\sum_{l,m} \boldsymbol{R}_n \boldsymbol{R}_m A_{n,m} = \frac{k}{2}\sum_{pq=0}^{N-1} \alpha_p^n \alpha_q^n \boldsymbol{u}_p \boldsymbol{u}_q, \tag{42}$$

where

$$A_{l,m} = 4\kappa \frac{2}{N}\sum_{p=0}^{N-1} \sin^\beta\left(\frac{p\pi}{2N}\right) \cos\left(\left(l - \frac{1}{2}\right)\frac{p\pi}{N}\right) \cos\left(\left(m - \frac{1}{2}\right)\frac{p\pi}{N}\right). \tag{43}$$

When a localized interaction acts on monomer n, the polymer energy becomes

$$\tilde{\phi}(\boldsymbol{R}_1, ..., \boldsymbol{R}_N) = \frac{1}{2}\sum_{l,m} \boldsymbol{R}_l \boldsymbol{R}_m A_{l,m} + \frac{1}{2}k\boldsymbol{R}_n^2, \tag{44}$$

which can be represented as

$$\tilde{\phi}(\boldsymbol{R}_1, ..., \boldsymbol{R}_N) = \frac{1}{2}\sum_{l,m} \boldsymbol{R}_l \boldsymbol{R}_m C_{l,m}, \tag{45}$$

where

$$C_{l,m} = \begin{cases} A_{n,n} + k, & \\ A_{l,m}, & \text{else.} \end{cases} \tag{46}$$

The probability distribution function is the Boltzmann distribution

$$P(\boldsymbol{R}) = \mathcal{N} e^{-\tilde{\phi}(\boldsymbol{R})}, \tag{47}$$

while the conditional probability distribution function is

$$P(\boldsymbol{R}|\boldsymbol{R}_c = \boldsymbol{x}) = \mathcal{N} e^{-\tilde{\phi}(\boldsymbol{R}_n,...,\boldsymbol{R}_{c-1},\boldsymbol{x},\boldsymbol{R}_{c+1})}. \tag{48}$$

The normalization factor $\mathcal{N}$ is given by

$$\begin{aligned}
\mathcal{N}^{-1} &= \int P(\boldsymbol{R}|\boldsymbol{R}_c = \boldsymbol{x}) \prod_{k\neq c} d\boldsymbol{R}_k = \int e^{-\tilde{\phi}(...,\boldsymbol{R}_{c-1},\boldsymbol{x},\boldsymbol{R}_{c+1},...)} \prod_{k\neq c} d\boldsymbol{R}_k \\
&= \\
&= \int e^{-\frac{1}{2}C_{c,c}\boldsymbol{x}^2} \exp\left[-\frac{1}{2}\left(\sum_{l,m\neq c} C_{l,m}\boldsymbol{R}_l\boldsymbol{R}_m + 2\sum_{l\neq c} C_{l,c}\boldsymbol{R}_l\boldsymbol{x}\right)\right] \\
&= \int e^{-\frac{1}{2}C_{c,c}\boldsymbol{x}^2} \exp\left[-\frac{1}{2}\sum_{l,m}\tilde{C}_{l,m}\boldsymbol{R}_l\boldsymbol{R}_m + \sum_l F_l\boldsymbol{R}_l\right] \\
&= \left[\frac{(2\pi)^{N-1}}{\det\tilde{\mathrm{C}}}\right]^{3/2} e^{-\frac{1}{2}B_{c,c}\boldsymbol{x}^2} e^{\frac{1}{2}\boldsymbol{F}^T\tilde{C}^{-1}\boldsymbol{F}},
\end{aligned} \tag{49}$$

The matrix $\tilde{C}$ is the reduced matrix C (eq.46) when the row and column c is removed. It is a block matrix with d blocks each of size $(N-1)\times(N-1)$, where each block i given by

$$\tilde{C}^i = \begin{pmatrix}
A_{1,1} & \cdots & \cdots & \cdots & & \\
\vdots & \vdots & \vdots & \vdots & \vdots & \vdots \\
\vdots & A_{n,n}+k & \vdots & \vdots & \vdots & \\
\vdots & \vdots & \ddots & \vdots & \vdots & \vdots \\
\vdots & \vdots & A_{c-1,c-1} & A_{c-1,c+1} & A_{c-1,c+2} & \vdots \\
\vdots & \vdots & A_{c+1,c-1} & A_{c+1,c+1} & A_{c+1,c+2} & \vdots \\
\vdots & \vdots & \vdots & \vdots & \vdots & \vdots
\end{pmatrix}, \tag{50}$$

and the vector $\boldsymbol{F}$ is compose of d blocks, each of size $(N-1)$:

$$F^i = \begin{pmatrix}
-A_{1,c}x^i \\
\vdots \\
-A_{c-1,c}x^i \\
-A_{c+1,c}x^i \\
\vdots \\
-A_{N,c}x^i
\end{pmatrix}. \tag{51}$$

The force acting on monomer c is given by

$$-\nabla_{\boldsymbol{R}_c}\tilde{\phi}(\boldsymbol{R}_1,...,\boldsymbol{R}_N) = -A_{c,c}\boldsymbol{R}_c - \sum_{l\neq c} A_{l,c}\boldsymbol{R}_l. \tag{52}$$

The conditional expectation of the velocity of monomer c at small time steps is

$$\lim_{\Delta t \to 0} \mathbb{E}\{\frac{\boldsymbol{R}_c(t+\Delta t) - \boldsymbol{R}_c(t)}{\Delta t} | \boldsymbol{R}_c = \boldsymbol{x}\} = D \int \left[-\nabla_{\boldsymbol{R}_c} \tilde{\phi}(\boldsymbol{R}_1, ..., \boldsymbol{R}_N) \right]_{\boldsymbol{R}_c = \boldsymbol{x}}$$
$$P(\boldsymbol{R}|\boldsymbol{R}_c = \boldsymbol{x}) \prod_{i \neq c} d\boldsymbol{R}_i, \tag{53}$$

where the conditional force is given by (52) and the conditional probability by (48) and (49). Thus

$$\lim_{\Delta t \to 0} \mathbb{E}\{\frac{\boldsymbol{R}_c(t+\Delta t) - \boldsymbol{R}_c(t)}{\Delta t} | \boldsymbol{R}_c = \boldsymbol{x}\} = D \int \left[-\nabla_{\boldsymbol{R}_c} \tilde{\phi}(\boldsymbol{R}_{c-1}, \boldsymbol{R}_c, \boldsymbol{R}_{c+1}) \right]_{\boldsymbol{R}_c = \boldsymbol{x}}$$
$$P(\boldsymbol{R}_{c-1}, \boldsymbol{R}_c, \boldsymbol{R}_{c+1} | \boldsymbol{R}_c = \boldsymbol{x}) \prod_{i \neq c} d\boldsymbol{R}_i =$$
$$-\mathcal{N} D e^{-\frac{1}{2} C_{c,c} \boldsymbol{x}^2} \int (A_{c,c} \boldsymbol{x} + \sum_{l \neq c} A_{l,c} \boldsymbol{R}_l) \exp \left[-\frac{1}{2} \sum_{l,m} \tilde{C}_{l,m} \boldsymbol{R}_l \boldsymbol{R}_m + \sum_l \boldsymbol{F}_l \boldsymbol{R}_l \right] \prod_{i \neq c} d\boldsymbol{R}_i =$$
$$-A_{c,c} D \boldsymbol{x} - \mathcal{N} D e^{-\frac{1}{2} C_{c,c} \boldsymbol{x}^2} \int \sum_{l \neq c} A_{l,c} \boldsymbol{R}_l \exp \left[-\frac{1}{2} \sum_{l,m} \tilde{C}_{l,m} \boldsymbol{R}_l \boldsymbol{R}_m + \sum_l \boldsymbol{F}_l \boldsymbol{R}_l \right] \prod_{i \neq c} d\boldsymbol{R}_i =$$
$$-A_{c,c} D \boldsymbol{x} - \mathcal{N} D e^{-\frac{1}{2} C_{c,c} \boldsymbol{x}^2} \left[\frac{(2\pi)^{n-1}}{\det \tilde{\mathrm{C}}} \right]^{3/2} \sum_{i, l \neq c} A_{l,c} \frac{\partial}{\partial F_l^i} e^{\frac{1}{2} \boldsymbol{F}^T \tilde{C}^{-1} \boldsymbol{F}} =$$
$$-A_{c,c} D \boldsymbol{x} - D e^{-\frac{1}{2} \boldsymbol{F}^T \tilde{C}^{-1} \boldsymbol{F}} \sum_{i, l \neq c} A_{l,c} \frac{\partial}{\partial F_l^i} e^{\frac{1}{2} \boldsymbol{F}_l \tilde{C}^{-1}_{l,m} \boldsymbol{F}_m} =$$
$$-A_{c,c} D \boldsymbol{x} - D \sum_{l \neq c} A_{l,c} \sum_{m \neq c} \tilde{C}^{-1}_{l,m} \boldsymbol{F}_m = -A_{c,c} D \boldsymbol{x} - D \sum_{l \neq c} A_{l,c} \sum_{m \neq c} \tilde{C}^{-1}_{l,m} (-A_{m,c} \boldsymbol{x}) =$$
$$-\boldsymbol{x} D \left(A_{c,c} - \sum_{l,m \neq c} A_{l,c} A_{m,c} \tilde{C}^{-1}_{l,k} \right). \tag{54}$$

We conclude that

$$\lim_{\Delta t \to 0} \mathbb{E}\{\frac{\boldsymbol{R}_c(t+\Delta t) - \boldsymbol{R}_c(t)}{\Delta t} | \boldsymbol{R}_c = \boldsymbol{x}\} = -\boldsymbol{x} D k_{cn}(\beta, N), \tag{55}$$

where

$$k_{cn}(\beta, N) = A_{c,c} - \sum_{l,m \neq c} A_{l,c} A_{m,c} \tilde{C}^{-1}_{l,k}. \tag{56}$$

While for a Rouse polymer the force depends only on the distance along the chain $|c - n|$, for a β-polymer, the effective spring coefficient depends on all monomers.

1 Extracting the strength of two potential wells for a Rouse polymer

For a Rouse polymer subjected to two gradient forces, the energy $U_{\text{ext}}(\boldsymbol{R})$ can now described by

$$\tilde{\Phi}(\boldsymbol{R}) = \frac{\kappa}{2}\sum_{j=2}^{N}(\boldsymbol{R}_j - \boldsymbol{R}_{j-1})^2 + \frac{1}{2}k_n(\boldsymbol{R}_n - \boldsymbol{\mu}_n)^2 + \frac{1}{2}k_m(\boldsymbol{R}_m - \boldsymbol{\mu}_m)^2. \tag{57}$$

The external potentials represent the interaction of two monomers. We compute here the average position $\langle \boldsymbol{R}_c \rangle$ of monomer c, which is situated between monomers n and m. For that goal, we rewrite the potential $\tilde{\Phi}(\boldsymbol{R})$ in a quadratic form

$$\tilde{\Phi}(\boldsymbol{R}) = \frac{1}{2}\sum_{p,q} A_{p,q}\boldsymbol{R}_p\boldsymbol{R}_q + \sum_p B_p\boldsymbol{R}_p + \frac{1}{2}k_n\boldsymbol{\mu}_n^2 + \frac{1}{2}k_m\boldsymbol{\mu}_m^2, \tag{58}$$

where the matrix A is made of d blocks, each of size $N \times N$, where each block i is given by

$$A^i = \begin{pmatrix} \kappa & -\kappa & 0 & \cdots & \cdots & \cdots & 0 \\ -\kappa & 2\kappa & -\kappa & \cdots & \vdots & \vdots & 0 \\ \vdots & \vdots & \vdots & \ddots & \vdots & \vdots & \\ 0 & \cdots & -\kappa & 2\kappa + k_n & -\kappa & 0 & \cdots \\ \vdots & \vdots & \vdots & \ddots & \vdots & \vdots & \\ \vdots & \vdots & 0 & -\kappa & 2\kappa + k_m & -\kappa & 0 \\ \vdots & \vdots & \vdots & \ddots & \vdots & \vdots & \vdots \\ 0 & 0 & 0 & \cdots & 0 & -\kappa & \kappa \end{pmatrix} \tag{59}$$

and the vector $\boldsymbol{B}$ is compose of d blocks, each given by

$$B^i = \begin{pmatrix} 0 \\ \vdots \\ k_n\mu_n \\ 0 \\ \vdots \\ k_m\mu_m \\ 0 \\ \vdots \end{pmatrix}. \tag{60}$$

Since A^i is a tridiagonal matrix, we use the algorithm proposed in [34] to calculate its determinant and inverse of matrix. For $a, b < m$, $A^i_{a,b}$ has the same structure as the matrix 7. Thus

$$\theta_l = \kappa^l \quad \text{for} \quad l < n, \tag{61}$$

$$\theta_n = \kappa^n + k_n \kappa^{n-1}, \tag{62}$$

$$\theta_l = \kappa^{l-1}\left(\kappa + (l-n+1)k_n\right), \quad \text{for } n \le l < m. \tag{63}$$

We next find the series solution at position m:

$$\theta_m = (2\kappa + k_m)\theta_{m-1} - \kappa^2\theta_{m-2} = \kappa^{m-2}\left[\kappa^2 + k_n\kappa(m-n+1) + k_m(\kappa + (m-n)k_n)\right] \tag{64}$$

and

$$\theta_{m+1} = 2\kappa\theta_m - \kappa^2\theta_{m-1} = \kappa^{m-1}\left[\kappa^2 + k_n\kappa(m-n+2) + 2k_m(\kappa + (m-n)k_n)\right]. \tag{65}$$

Again, we solve the recurrence relation

$$\begin{aligned}
&\theta_l = 2\kappa\theta_{l-1} - \kappa^2\theta_{l-2}, \quad \text{for } m+1 < l < N,\\
&\theta_m = (2\kappa + k_m)\theta_{m-1} - \kappa^2\theta_{m-2} = \kappa^{m-2}\left[\kappa^2 + k_n\kappa(m-n+1) + k_m(\kappa + (m-n)k_n)\right],\\
&\theta_{m+1} = 2\kappa\theta_m - \kappa^2\theta_{m-1} = \kappa^{m-1}\left[\kappa^2 + k_n\kappa(m-n+2) + 2k_m(\kappa + (m-n)k_n)\right].
\end{aligned} \tag{66}$$

Eq.(66) has the characteristic polynomial (eq.12), thus using the initial conditions (eq.66), we find

$$\theta_l = \kappa^{l-2}\left(\kappa^2 + \kappa(k_n(l-n+1) + k_m(l-m+1)) + k_n k_m(m-n)(l+1-m)\right) \quad \text{for } m \le l < N. \tag{67}$$

Using that

$$\theta_N = \kappa\theta_{N-1} - \kappa^2\theta_{N-2}, \tag{68}$$

we obtain that the determinant is given by

$$\det A^i = \theta_N = \kappa^{N-2}\left((k_m + k_n)\kappa + (m-n)k_n k_m\right). \tag{69}$$

The inverse of the matrix A^i is given by [34]

$$(A^i)^{-1}_{ij} = \begin{cases} (-1)^{i+j} c_i c_{i+1} ... c_{j-1} \frac{\theta_{i-1}\phi_{j+1}}{\theta_N}, \ i \le j \\ (-1)^{i+j} a_j a_{j+1} ... a_{i-1} \frac{\theta_{j-1}\phi_{i+1}}{\theta_N}, \ i > j \\ \frac{\theta_{i-1}\phi_{i+1}}{\theta_N} \quad i = j, \end{cases} \tag{70}$$

where

$$\begin{aligned}
&\phi_l = b_l\theta_{l+1} - a_{l+1}c_l\theta_{l+2} \quad \text{for } l \le N,\\
&\phi_{N+1} = 1,\\
&\phi_{N+2} = 0.
\end{aligned} \tag{71}$$

Solving the recurrence relation 71 we find

$$\begin{aligned}
&\phi_l = \kappa^{N-l+1} \quad \text{for } m < l < N,\\
&\phi_l = \kappa^{-m-1}\left[\kappa^{N+m-l+2} + (m-l+1)k_m\kappa^{N+m-l+1}\right] \quad \text{for } n < l \le m,\\
&\phi_n = \kappa^{N-n-1}\left[\kappa^2 + k_m\kappa(m-n+1) + k_n(\kappa + (m-n)k_m)\right].
\end{aligned} \tag{72}$$

The normalization factor is

$$
\begin{aligned}
\mathcal{N}^{-1} &= \int_\Omega .. \int_\Omega \prod_{i=1}^{N} d\boldsymbol{R}_i P(\boldsymbol{R}) = \int_\Omega .. \int_\Omega \prod_{i=1}^{N} d\boldsymbol{R}_i \exp\Big(-\frac{1}{2}\sum_{p,q} A_{p,q}\boldsymbol{R}_p\boldsymbol{R}_q \\
&+ \sum_p B_p \boldsymbol{R}_p + \frac{1}{2}k(\boldsymbol{\mu}_n^2 + \boldsymbol{\mu}_m^2)\Big) = \left[\frac{(2\pi)^N}{\prod_i \det \mathrm{A^i}}\right]^{3/2} e^{-\frac{1}{2}k(\boldsymbol{\mu}_n^2+\boldsymbol{\mu}_m^2)} e^{\frac{1}{2}\boldsymbol{B}^T A^{-1}\boldsymbol{B}} \quad (73)
\end{aligned}
$$

Substituting eq.(60) in the exponential term of the last equation, we get

$$
\boldsymbol{B}^T A^{-1}\boldsymbol{B} = \sum_i (k\mu_n^i)^2 (A^i)^{-1}_{nn} + (k\mu_m^i)^2 (A^i)^{-1}_{mm} + k^2\mu_n^i\mu_m^i (A^i)^{-1}_{nm} + k^2\mu_n^i\mu_m^i (A^i)^{-1}_{mn}. \quad (74)
$$

We now estimate these elements of $(A^i)^{-1}$:

$$
\begin{aligned}
(A^i)^{-1}_{nn} &= \frac{\theta_{n-1}\phi_{n+1}}{\theta_N} = \frac{\kappa + k_m(m-n)}{k_n k_m (m-n) + (k_n+k_m)\kappa}, \\
(A^i)^{-1}_{mm} &= \frac{\theta_{m-1}\phi_{m+1}}{\theta_N} = \frac{\kappa + k_n(m-n)}{k_n k_m (m-n) + (k_n+k_m)\kappa}, \\
(A^i)^{-1}_{nm} &= (-1)^{n+m}(-\kappa)^{m-n}\frac{\theta_{n-1}\phi_{m+1}}{\theta_N} = \frac{\kappa}{k_n k_m (m-n) + (k_n+k_m)\kappa} \quad (75)
\end{aligned}
$$

Finally, we compute the average position of monomer c

$$
\begin{aligned}
\langle \boldsymbol{R}_c \rangle &= \int_\Omega .. \int_\Omega \prod_{i=1}^{N} d\boldsymbol{R}_i \boldsymbol{R}_c P(\boldsymbol{R}) = \mathcal{N}\int_\Omega .. \int_\Omega \prod_{i=1}^{N} d\boldsymbol{R}_i \boldsymbol{R}_c \exp\Big(-\frac{1}{2}\sum_{p,q} A_{p,q}\boldsymbol{R}_p\boldsymbol{R}_q \\
&+ \sum_p B_p\boldsymbol{R}_p + \frac{1}{2}k_n\boldsymbol{\mu}_n^2 + \frac{1}{2}k_m\boldsymbol{\mu}_m^2\Big) = \sum_i k_n\mu_n^i (A^i)^{-1}_{cn} + k_m\mu_m^i (A^i)^{-1}_{cm}, \quad (76)
\end{aligned}
$$

where

$$
\begin{aligned}
(A^i)^{-1}_{cn} &= (-1)^{c+n}(-\kappa)^{c-n}\frac{\theta_{n-1}\phi_{c+1}}{\theta_N} = \frac{\kappa + (m-c)k_m}{k_n k_m (m-n) + (k_n+k_m)\kappa}, \\
(A^i)^{-1}_{cm} &= (-1)^{c+m}(-\kappa)^{m-c}\frac{\theta_{c-1}\phi_{m+1}}{\theta_N} = \frac{\kappa + (c-n)k_n}{k_n k_m (m-n) + (k_n+k_m)\kappa}. \quad (77)
\end{aligned}
$$

Thus

$$
\langle \boldsymbol{R}_c \rangle = \frac{1}{k_n k_m (m-n) + (k_n+k_m)\kappa}\sum_i \mu_n^i k_n(\kappa + (m-c)k_m) + \mu_m^i k_m(\kappa + (c-n)k_n). \quad (78)
$$

We now estimate the conditional expectation of the velocity for the observed monomer c, in the small time step regime

$$
\lim_{\Delta t \to 0} \mathbb{E}\{\frac{\boldsymbol{R}_c(t+\Delta t) - \boldsymbol{R}_c(t)}{\Delta t} | \boldsymbol{R}_c = \boldsymbol{x}\}. \quad (79)
$$

The conditional expectation of the velocity of monomer c is

$$\lim_{\Delta t\to 0}\mathbb{E}\{\frac{\boldsymbol{R}_c(t+\Delta t)-\boldsymbol{R}_c(t)}{\Delta t}|\boldsymbol{R}_c=\boldsymbol{x}\} = D\int\left[-\nabla_{\boldsymbol{R}_c}\tilde{\Phi}(\boldsymbol{R})\right]_{\boldsymbol{R}_c=\boldsymbol{x}} P(\boldsymbol{R}|\boldsymbol{R}_c=\boldsymbol{x})\prod_i d\boldsymbol{R}_i$$

$$= D\int\left(\kappa\left(\boldsymbol{x}-\boldsymbol{R}_{c-1}\right)-\kappa\left(\boldsymbol{R}_{c+1}-\boldsymbol{x}\right)\right)P(\boldsymbol{R}|\boldsymbol{R}_c=\boldsymbol{x})\prod_i d\boldsymbol{R}_i = -\tilde{\mathcal{N}}De^{-\kappa\boldsymbol{x}^2-\frac{1}{2}k_n\boldsymbol{\mu}_n^2-\frac{1}{2}k_m\boldsymbol{\mu}_m^2}$$

$$\int\left(\kappa\left(\boldsymbol{x}-\boldsymbol{R}_{c-1}\right)-\kappa\left(\boldsymbol{R}_{c+1}-\boldsymbol{x}\right)\right)e^{-\frac{1}{2}\sum_{i,p,q}\tilde{A}^i_{p,q}R_pR_q+\sum_{i,p}\tilde{B}^i_pR^i_p}\prod_{i\neq c}d\boldsymbol{R}_i$$

$$= -D\kappa\sum_i\left(2x^i-\sum_l\tilde{B}^i_l\left((\tilde{A}^i)^{-1}_{c-1,l}+(\tilde{A}^i)^{-1}_{c+1,l}\right)\right), \tag{80}$$

where $\tilde{\mathcal{N}}$ is the normalization as computed in 73 when the c column is removed. $\tilde{A}^i$ is a block matrix of size $N-1\times N-1$

$$\tilde{A}^i = \begin{pmatrix}\tilde{A}^i_1 & 0\\ 0 & \tilde{A}^i_2\end{pmatrix} \tag{81}$$

with

$$\tilde{A}^i_1 = \begin{pmatrix}\kappa & -\kappa & 0 & \cdots & 0 & 0\\ -\kappa & 2\kappa & -\kappa & \cdots & 0 & 0\\ \vdots & \vdots & \vdots & \ddots & \vdots & \vdots\\ \vdots & 0 & -\kappa & 2\kappa+k_n & -\kappa & \vdots\\ \vdots & \vdots & \vdots & \ddots & \vdots & \vdots\\ \vdots & 0 & 0 & -\kappa & 2\kappa & -\kappa\\ \vdots & 0 & 0 & 0 & -\kappa & 2\kappa\end{pmatrix} \tag{82}$$

which is of size $(c-1)\times(c-1)$ and

$$\tilde{A}^i_2 = \begin{pmatrix}2\kappa & -\kappa & 0 & \cdots & 0 & 0\\ -\kappa & 2\kappa & -\kappa & \cdots & 0 & 0\\ \vdots & \vdots & \vdots & \ddots & \vdots & \vdots\\ \vdots & 0 & -\kappa & 2\kappa+k_m & -\kappa & \vdots\\ \vdots & \vdots & \vdots & \ddots & \vdots & \vdots\\ \vdots & 0 & 0 & -\kappa & 2\kappa & -\kappa\\ \vdots & 0 & 0 & 0 & -\kappa & \kappa\end{pmatrix} \tag{83}$$

which is of size $(N-c)\times(N-c)$. The vector $\tilde{\boldsymbol{B}}$ is compose of d blocks, each given by

$$\tilde{B}^i = \begin{pmatrix} 0 \\ \vdots \\ k_n\mu_n \\ \vdots \\ \kappa x^i \\ \kappa x^i \\ \vdots \\ k_m\mu_m \\ \vdots \end{pmatrix}. \tag{84}$$

To find the determinant $\det\tilde{A}_1^i$, we use eq.(63):

$$\det\tilde{A}_1^i = \kappa^{c-2}(\kappa + (c-n)k_n) \tag{85}$$

and using eq.(72)

$$\det\tilde{A}_2^i = \kappa^{N-c-1}(\kappa + (m-c)k_m). \tag{86}$$

The elements of the inverse matrix are given by

$$\begin{aligned}
(\tilde{A_1}^i)^{-1}_{c-1,c-1} &= \frac{\kappa+(c-n-1)k_n}{\kappa^2+(c-n)k_n\kappa} = \frac{1}{\kappa} - \frac{k_n}{\kappa(\kappa+(c-n)k_n)}, \\
(\tilde{A_2}^i)^{-1}_{c+1,c+1} &= \frac{\kappa+(m-c-1)k_m}{\kappa^2+(m-c)k_m\kappa} = \frac{1}{\kappa} - \frac{k_m}{\kappa(\kappa+(m-c)k_m)}, \\
(\tilde{A_1}^i)^{-1}_{c-1,n} &= (-1)^{c+n-1}(-\kappa)^{c-n-1}\frac{\theta_{n-1}}{\theta_{c-1}} = \frac{1}{\kappa+(c-n)k_n}, \\
(\tilde{A_2}^i)^{-1}_{c+1,m} &= \frac{1}{\kappa+(m-c)k_m}.
\end{aligned} \tag{87}$$

Now, we estimate the term in the parenthesis in eq.(80) by substituting eqs.(87)

$$\begin{aligned}
K^i &= 2x^i - \sum_l \tilde{B}_l^i \left((\tilde{A}^i)^{-1}_{c-1,l} + (\tilde{A}^i)^{-1}_{c+1,l} \right) \\
&= 2x^i - \left(\kappa x^i (\tilde{A_1}^i)^{-1}_{c-1,c-1} + \kappa x^i (\tilde{A_1}^i)^{-1}_{c+1,c+1} + k_n\mu_n (\tilde{A_1}^i)^{-1}_{c-1,n} + k_m\mu_m (\tilde{A_2}^i)^{-1}_{c+1,n} \right) \\
&= 2x^i - \kappa x^i \left(\frac{\kappa+(c-n-1)k_n}{\kappa^2+(c-n)k_n\kappa} + \frac{\kappa+(m-c-1)k_m}{\kappa^2+(m-c)k_m\kappa} \right) - \frac{k_n\mu_n^i}{\kappa+(c-n)k_n} - \frac{k_m\mu_m^i}{\kappa+(m-c)k_m} \\
&= 2x^i - x^i \left(\frac{\kappa+(c-n-1)k_n}{\kappa+(c-n)k_n} + \frac{\kappa+(m-c-1)k_m}{\kappa+(m-c)k_m} \right) - \frac{k_n\mu_n^i}{\kappa+(c-n)k_n} - \frac{k_m\mu_m^i}{\kappa+(m-c)k_m} \\
&= 2x^i - x^i \left(2 - \frac{k_n}{\kappa+(c-n)k_n} - \frac{k_m}{\kappa+(m-c)k_m} \right) - \frac{k_n\mu_n^i}{\kappa+(c-n)k_n} - \frac{k_m\mu_m^i}{\kappa+(m-c)k_m} \\
&= x^i \left(\frac{k_n}{\kappa+(c-n)k_n} + \frac{k_m}{\kappa+(m-c)k_m} \right) - \frac{k_n\mu_n^i}{\kappa+(c-n)k_n} - \frac{k_m\mu_m^i}{\kappa+(m-c)k_m}
\end{aligned} \tag{88}$$

We introduce into expression eq.(88) the position of monomer c with respect to its mean position ($\tilde{\boldsymbol{x}} = \boldsymbol{x} - \langle \boldsymbol{R}_c \rangle$) (eq.78)

$$
\begin{aligned}
K^i &= \left[\tilde{x^i} + \frac{1}{k_n k_m (m-n) + (k_n + k_m)\kappa} \sum_i \mu_n^i k_n (\kappa + (m-c)k_m) + \mu_m^i k_m (\kappa + (c-n)k_n) \right] \\
&\quad \left(\frac{k_n}{\kappa + (c-n)k_n} + \frac{k_m}{\kappa + (m-c)k_m} \right) - \frac{k_n \mu_n^i}{\kappa + (c-n)k_n} - \frac{k_m \mu_m^i}{\kappa + (m-c)k_m} \\
&= \tilde{x^i} \left(\frac{k_n}{\kappa + (c-n)k_n} + \frac{k_m}{\kappa + (m-c)k_m} \right). \qquad (89)
\end{aligned}
$$

Finally, substituting eq.(89) into expression (80), we find

$$
\lim_{\Delta t \to 0} \mathbb{E}\{ \frac{\boldsymbol{R}_c(t+\Delta t) - \boldsymbol{R}_c(t)}{\Delta t} | \tilde{\boldsymbol{x}} \} = -D(k_{c,n} + k_{c,m}) \tilde{\boldsymbol{x}}, \qquad (90)
$$

where

$$
k_{c,n} = \frac{k_n \kappa}{\kappa + (c-n)k_n} \qquad (91)
$$

and

$$
k_{c,m} = \frac{k_m \kappa}{\kappa + (m-c)k_m}. \qquad (92)
$$

1.1 Variance of monomer c position

We now find the variance of a monomer position $\langle \boldsymbol{R}_c^2 \rangle$ with respect to its average position

$$
\langle (\boldsymbol{R}_c - \langle \boldsymbol{R}_c \rangle)^2 \rangle = \langle \boldsymbol{R}_c^2 \rangle - \langle \boldsymbol{R}_c \rangle^2, \qquad (93)
$$

We begin by calculating

$$
\begin{aligned}
\langle \boldsymbol{R}_c^2 \rangle &= \int_\Omega .. \int_\Omega d\boldsymbol{R}_1 d\boldsymbol{R}_2 ... d\boldsymbol{R}_N \boldsymbol{R}_c^2 P(\boldsymbol{R}) = \mathcal{N} \left[\frac{(2\pi)^{d(N-1)}}{\det A} \right]^{1/2} \sum_i \frac{\partial^2}{\partial (R_c^i)^2} \exp \left(\frac{1}{2} \sum_i \sum_{pq} A^{i\,-1}_{pq} \right) \\
&= \sum_i (A^i)^{-1}_{cc} + \left((A^i)^{-1}_{cn} k_n \mu_n^i + (A^i)^{-1}_{cm} k_m \mu_m^i \right)^2, \qquad (94)
\end{aligned}
$$

where

$$
(A^i)^{-1}_{cc} = \frac{\theta_{c-1}\phi_{c+1}}{\theta_N} = \frac{(\kappa + (c-n)k_n)(\kappa + (m-c)k_n)}{(k_m + k_n)\kappa^2 + (m-n)k_n k_m \kappa}. \qquad (95)
$$

Substituting (94), (95) and (78) into eq.(93) we find

$$
\langle (\boldsymbol{R}_c - \langle \boldsymbol{R}_c \rangle)^2 \rangle = \frac{1}{k_{cn} + k_{cm}}. \qquad (96)
$$

1.2 Two potential wells located on the left side of the observed locus ($n < m < c$)

Finally, we solve for the case $n < m < c$. We being with the average position of monomer c

$$\langle \boldsymbol{R}_c \rangle = \sum_i k_n \mu_n^i (A^i)^{-1}_{cn} + k_m \mu_m^i (A^i)^{-1}_{cm}, \tag{97}$$

where A^i is given in eq.(59). Thus

$$\begin{aligned} (A^i)^{-1}_{cn} &= (-1)^{c+n}(-\kappa)^{c-n}\frac{\theta_{n-1}\phi_{c+1}}{\theta_N} = \frac{\kappa}{k_n k_m(m-n)+\kappa(k_n+k_m)}, \\ (A^i)^{-1}_{cm} &= (-1)^{c+m}(-\kappa)^{m-c}\frac{\theta_{m-1}\phi_{c+1}}{\theta_N} = \frac{\kappa+(m-n)k_n}{k_n k_m(m-n)+\kappa(k_n+k_m)}. \end{aligned} \tag{98}$$

The average position of c is

$$\langle \boldsymbol{R}_c \rangle = \frac{1}{k_n k_m(m-n)+\kappa(k_n+k_m)} \sum_i k_n \mu_n^i \kappa + k_m \mu_m^i (\kappa + (m-n)k_n). \tag{99}$$

The conditional expectation of the velocity of monomer c for a small time step is defined as

$$\begin{aligned} \lim_{\Delta t \to 0} \mathbb{E}\{\frac{\boldsymbol{R}_c(t+\Delta t)-\boldsymbol{R}_c(t)}{\Delta t}|\boldsymbol{x}\} &= D \int \left[-\nabla_{\boldsymbol{R}_c}\tilde{\Phi}(\boldsymbol{R})\right]_{\boldsymbol{R}_c=\boldsymbol{x}} P(\boldsymbol{R}|\boldsymbol{R}_c=\boldsymbol{x}) \prod_i d\boldsymbol{R}_i \\ &= -D\kappa \sum_i \left(2x^i - \sum_l V_l^i \left((U^i)^{-1}_{c-1,l} + (U^i)^{-1}_{c+1,l}\right)\right), \end{aligned} \tag{100}$$

where U^i is also a block matrix of size $N-1 \times N-1$

$$U^i = \begin{pmatrix} U_1^i & 0 \\ 0 & U_2^i \end{pmatrix} \tag{101}$$

with

$$U_1^i = \begin{pmatrix} \kappa & -\kappa & 0 & \cdots & 0 & 0 & 0 \\ -\kappa & 2\kappa & -\kappa & \cdots & 0 & 0 & 0 \\ \vdots & \vdots & \vdots & \ddots & \vdots & \vdots & \vdots \\ \vdots & 0 & -\kappa & 2\kappa+k_n & -\kappa & 0 & \vdots & \vdots \\ \vdots & \vdots & \vdots & \ddots & \vdots & \vdots & \vdots \\ \vdots & 0 & 0 & -\kappa & 2\kappa+k_m & -\kappa & \vdots \\ \vdots & \vdots & \vdots & \ddots & \vdots & \vdots & \vdots \\ \vdots & 0 & 0 & 0 & -\kappa & 2\kappa & -\kappa \\ \vdots & 0 & 0 & 0 & 0 & -\kappa & 2\kappa \end{pmatrix} \tag{102}$$

which is of size $(c-1)\times(c-1)$ and

$$U_2^i = \begin{pmatrix} 2\kappa & -\kappa & 0 & \cdots & 0 & 0 \\ -\kappa & 2\kappa & -\kappa & \cdots & 0 & 0 \\ \vdots & \vdots & \vdots & \ddots & \vdots & \vdots \\ \vdots & 0 & 0 & -\kappa & 2\kappa & -\kappa \\ \vdots & 0 & 0 & 0 & -\kappa & \kappa \end{pmatrix} \tag{103}$$

which is of size $(N-c)\times(N-c)$. The vector $\boldsymbol{V}$ is composed of d blocks, each given by

$$V^i = \begin{pmatrix} 0 \\ \vdots \\ k_n\mu_n \\ \vdots \\ k_m\mu_m \\ \vdots \\ \kappa x^i \\ \kappa x^i \\ \vdots \end{pmatrix}. \tag{104}$$

To find the determinant $\det\tilde{U}_1^i$, we use eq.(67):

$$\det U_1^i = \theta_{c-1} = \kappa^{c-3}\left(\kappa^2 + ((c-n)k_n + (c-m)k_m)\kappa + (m-n)(c-m)k_nk_m\right) \tag{105}$$

and using eq.(61), we get

$$\det U_2^i = \kappa^{N-c}. \tag{106}$$

The (c-1,c-1) term of the inverse matrix is given by

$$\begin{aligned} (U^i)^{-1}_{c-1,c-1} &= \frac{\theta_{c-2}\phi_{c+1}}{\det(U_2^i)\det(U_1^i)} \\ &= \frac{\kappa^2 + ((c-n-1)k_n + (c-m-1)k_m)\kappa + (m-n)(c-m-1)k_nk_m}{\kappa\left(\kappa^2 + ((c-n)k_n + (c-m)k_m)\kappa + (m-n)(c-m)k_nk_m\right)} \\ &= \frac{1}{\kappa} - \frac{\kappa(k_n+k_m) + (m-n)k_nk_m}{\kappa\left(\kappa^2 + ((c-n)k_n + (c-m)k_m)\kappa + (m-n)(c-m)k_nk_m\right)}, \end{aligned} \tag{107}$$

where we used eqs.(72),(67). For simplicity we name the indices of the matrix U $(i = 1, 2..c-1, c+1, ..N)$

$$(U^i)^{-1}_{c+1,c+1} = \frac{\theta_{c-1}\phi_{c+2}}{\det(U_2^i)\det(U_1^i)} = \frac{1}{\kappa}, \tag{108}$$

$$(U^i)^{-1}_{c-1,n} = \frac{(-1)^{c+n-1}(-\kappa)^{c-n-1}\theta_{n-1}}{\det(U^i_1)} = \frac{\kappa}{\kappa^2 + ((c-n)k_n + (c-m)k_m)\kappa + (m-n)(c-m)k_n k_m} \quad (109)$$

and

$$(U^i)^{-1}_{c-1,m} = \frac{(-1)^{c+m-1}(-\kappa)^{c-m-1}\theta_{m-1}}{\det(U^i_1)} = \frac{\kappa + (m-n)k_n}{\kappa^2 + ((c-n)k_n + (c-m)k_m)\kappa + (m-n)(c-m)k_n k_m} \quad (110)$$

We estimate the term in the parenthesis in eq.(100) by substituting eqs.(107)-(110)

$$\begin{aligned}
J^i &= 2x^i - \sum_l V^i_l \left((U^i)^{-1}_{c-1,l} + (U^i)^{-1}_{c+1,l}\right) \\
&= 2x^i - \left(\kappa x^i (U^i)^{-1}_{c-1,c-1} + \kappa x^i (U^i)^{-1}_{c+1,c+1} + k_n \mu_n (U^i)^{-1}_{c-1,n} + k_m \mu_m (U^i)^{-1}_{c+1,n}\right) \\
&= 2x^i - \kappa x^i \left(\frac{2}{\kappa} - \frac{(k_n + k_m)\kappa + (m-n)k_n k_m}{\kappa\left(\kappa^2 + ((c-n)k_n + (c-m)k_m)\kappa + (m-n)(c-m)k_n k_m\right)}\right) \\
&- \frac{\kappa k_n \mu^i_n + (\kappa + (m-n)k_n)k_m \mu^i_m}{\kappa^2 + ((c-n)k_n + (c-m)k_m)\kappa + (m-n)(c-m)k_n k_m} \\
&= x^i \left(\frac{(k_n + k_m)\kappa + (m-n)k_n k_m}{\kappa^2 + ((c-n)k_n + (c-m)k_m)\kappa + (m-n)(c-m)k_n k_m}\right) \\
&- \frac{\kappa k_n \mu^i_n + (\kappa + (m-n)k_n)k_m \mu^i_m}{\kappa^2 + ((c-n)k_n + (c-m)k_m)\kappa + (m-n)(c-m)k_n k_m}. \qquad (111)
\end{aligned}$$

We introduce into expression eq.(111) the position of monomer c with respect to its mean position ($\tilde{\boldsymbol{x}} = \boldsymbol{x} - \langle R_c \rangle$) (eq.99)

$$\begin{aligned}
J^i &= \left[\tilde{x^i} + \frac{1}{k(m-n) + 2\kappa} \sum_i \mu^i_n \kappa + \mu^i_m (\kappa + (m-n)k)\right] \\
&\times \left(\frac{(k_n + k_m)\kappa + (m-n)k_n k_m}{\kappa^2 + ((c-n)k_n + (c-m)k_m)\kappa + (m-n)(c-m)k_n k_m}\right) \\
&- \frac{\kappa k_n \mu^i_n + (\kappa + (m-n)k_n)k_m \mu^i_m}{\kappa^2 + ((c-n)k_n + (c-m)k_m)\kappa + (m-n)(c-m)k_n k_m} \\
&= \tilde{x^i} \frac{(k_n + k_m)\kappa + (m-n)k_n k_m}{\kappa^2 + ((c-n)k_n + (c-m)k_m)\kappa + (m-n)(c-m)k_n k_m}. \qquad (112)
\end{aligned}$$

Finally, substituting eq.(112) into expression (100) we find

$$\lim_{\Delta t \to 0} \mathbb{E}\left\{\frac{\boldsymbol{R}_c(t + \Delta t) - \boldsymbol{R}_c(t)}{\Delta t} | \tilde{\boldsymbol{x}}\right\} = -D k_{c,nm} \tilde{\boldsymbol{x}}, \qquad (113)$$

where

$$k_{c,nm} = \kappa \frac{\kappa(k_n + k_m) + (m-n)k_n k_m}{\kappa^2 + ((c-n)k_n + (c-m)k_m)\kappa + (m-n)(c-m)k_n k_m}. \qquad (114)$$

2 Computing the auto-correlation function of the tagged locus when a force is applied

We now study the effect of the potential well on the second moment of the locus dynamics by calculating its auto-correlation function. The external potential can be written in term of the modes $\boldsymbol{u}_p$

$$U_{\text{ext}}(\boldsymbol{R}_n) = \frac{1}{2}k(\boldsymbol{\mu} - \boldsymbol{R}_n)^2 = \frac{1}{2}k\left(\boldsymbol{\mu} - \sum_{p=0}^{N-1}\alpha_p^n \boldsymbol{u}_p\right)^2, \tag{115}$$

where we used the orthogonal transformation which diagonalize the Rouse potential [21]

$$\alpha_p^c = \begin{cases} \sqrt{\frac{1}{N}}, & p = 0 \\ \sqrt{\frac{2}{N}}\cos\left((c - 1/2)\frac{p\pi}{N}\right), & \text{otherwise.} \end{cases} \tag{116}$$

In the presence of the potential, the Langevin equations for the modes are

$$\frac{d\boldsymbol{u}_p}{dt} = D\left(k\alpha_p^n\boldsymbol{\mu} - ((\alpha_p^n)^2 k + \tilde{\kappa}_p)\boldsymbol{u}_p\right) - Dk\alpha_p^n \sum_{q=0, q\neq p}^{N-1} \alpha_q^n \boldsymbol{u}_q + \sqrt{2D}\frac{d\tilde{\boldsymbol{w}}_p}{dt}, \tag{117}$$

with $\tilde{\kappa}_p = 4\kappa \sin\left(\frac{p\pi}{2N}\right)^\beta$ ($\beta > 1$) [24]. Thus, the potential on monomer n couples the dynamical equations for the modes. When the strength of the coupling term is relatively weak $(\alpha_p^n)^2 k \ll \tilde{\kappa}_p$, we can neglect the coupling term. This will be the case for the higher modes given that $k < \kappa$ and N large. In this case, the Langevin equations 117 can be approximated by

$$\begin{aligned} \frac{d\boldsymbol{u}_p}{dt} &= D\left(k\alpha_p^n\boldsymbol{\mu} - ((\alpha_p^n)^2 k + \tilde{\kappa}_p)\boldsymbol{u}_p\right) + \sqrt{2D}\frac{d\tilde{\boldsymbol{w}}_p}{dt}, \\ \frac{d\boldsymbol{u}_0}{dt} &= D_{\text{cm}}\alpha_0^n\left(\boldsymbol{\mu} - \alpha_0^n\boldsymbol{u}_0\right) + \sqrt{2D_{\text{cm}}}\frac{d\tilde{\boldsymbol{w}}_0}{dt}. \end{aligned} \tag{118}$$

We denote $b_p = (\alpha_p^n)^2 k + \tilde{\kappa}_p$. The solutions of the eqs.(118) is

$$\boldsymbol{u}_p(t) = \boldsymbol{u}_p(0)e^{-Db_p t} + \frac{k\alpha_p^n\boldsymbol{\mu}}{b_p}\left(1 - e^{-Db_p t}\right) + \sqrt{2D_p}\int_0^t e^{Db_p(s-t)}d\tilde{\boldsymbol{w}}_p(s), \tag{119}$$

while the expectation values are

$$\begin{aligned} \langle\boldsymbol{u}_p(t)\rangle &= \boldsymbol{u}_p(0)e^{-Db_p t} + \frac{k\alpha_p^n\boldsymbol{\mu}}{b_p}\left(1 - e^{-Db_p t}\right), \\ \langle\boldsymbol{u}_0(t)\rangle &= \boldsymbol{u}_0(0)e^{-D_{\text{cm}}k(\alpha_0^n)^2 t} + \frac{\boldsymbol{\mu}}{\alpha_0^n}\left(1 - e^{-D_{\text{cm}}k\alpha_0^n t}\right). \end{aligned} \tag{120}$$

The time auto-correlation function of mode p in the spatial direction i is [26]

$$\begin{aligned} &\left\langle\left[u_p^i(t_1) - \langle u_p^i(t_1)\rangle\right]\left[u_p^i(t_2) - \langle u_p^i(t_2)\rangle\right]\right\rangle = 2D\left\langle\int_0^{t_1} e^{Db_p(s_1-t_1)}d\omega_p^i(s_1)\int_0^{t_2} e^{Db_p(s_2-t_2)}d\omega_p^i(s_2)\right\rangle \\ &= \frac{1}{b_p}\left(e^{-Db_p(t_2-t_1)} - e^{-Db_p(t_1+t_2)}\right) \approx \frac{1}{(\alpha_p^n)^2 k + \tilde{\kappa}_p}e^{-D((\alpha_p^n)^2 k + \tilde{\kappa}_p)(t_2-t_1)}, \end{aligned} \tag{121}$$

for $t_2 > t_1$, where we approximated for long times and introduced back the expression for b_p. Similarly, for the center of mass we have

$$\langle \left[u_0^i(t_1) - \langle u_0^i(t_1)\rangle\right] \left[u_0^i(t_2) - \langle u_0^i(t_2)\rangle\right]\rangle \approx \frac{1}{(\alpha_0^n)^2 k} e^{-D_{\rm cm}(\alpha_0^n)^2 k(t_2-t_1)}. \quad (122)$$

Thus, the auto-correlation function of monomer c is

$$\langle[\boldsymbol{R}_c(t_1) - \langle\boldsymbol{R}_c(t_1)\rangle]\,[\boldsymbol{R}_c(t_2) - \langle\boldsymbol{R}_c(t_2)\rangle]\rangle = \frac{d}{k} e^{-D(\alpha_0^n)^2 k(t_2-t_1)} + \sum_{p=1}^{N-1} \frac{d(\alpha_p^c)^2}{(\alpha_p^n)^2 k + \tilde{\kappa}_p} e^{-D((\alpha_p^n)^2 k+\tilde{\kappa}_p)(t_2-t_1)}, \quad (123)$$

where $(\alpha_0^n)^2 = \frac{1}{N}$.

References

[1] R. Metzler and J. Klafter, Phys. Rep. **339**, 1 (2000).

[2] E. Kepten, I. Bronshtein and Y. Garini, Phys. Rev. E **83**, 041919 (2011).

[3] H. Wong, H. Marie-Nelly, S. Herbert, P. Carrivain, H. Blanc, R. Koszul, E. Fabre and C. Zimmer, Curr. Biol. **22**, 1881-1890 (2012).

[4] N. Tokuda, T. P. Terada and M. Sasai, Biophys J **102**, 296-304 (2012).

[5] I. M. Sokolov, Phys. Rev. Lett. **90**, 080601 (2003).

[6] Y. Kantor and M. Kardar, Phys. Rev. E **76**, 061121 (2007).

[7] S. E. Lee, J. K. Moore, A. Holmes, K. Umezu, R. D. Kolodner, J. E. Haber, Cell **94**, 399-409 (1998).

[8] P. Meister, L. R. Gehlen, E. Varela, V. Kalck and S. M. Gasser, Methods Enzymol. **470**, 535-567 (2010).

[9] C. Zimmer and E. Fabre, J. Cell Biol. **192**, 723-733 (2011).

[10] G. Fudenberg and L. A. Mirny, Curr Opin Genet Dev. **22**, 115-24 (2012).

[11] J. Dekker, M. A. Marti-Renom, L. A. Mirny, Nat Rev Genet. **14**, 390-403 (2013).

[12] V. Dion, V. Klack, C. Horigome, B. D. Towbin and S. M. Gasser, Nat. Cell Biol. **14**, 502-509 (2012).

[13] J. Mine-Hattab and R. Rothstein, Nat. Cell Biol. **14**, 510-517 (2012).

[14] S. C. Weber, M. A. Thompson, W. Moerner, A. J. Spakowitz and J. A. Theriot, Biophys. J. **102**, 2443 (2013).

[15] G. Hummer and A. Szabo, Free energy profiles from single-molecule pulling experiments. Proc Natl Acad Sci U S A, **107**, 21441-6 (2010).

[16] G. Hummer and A. Szabo, Kinetics from nonequilibrium single-molecule pulling experiments. Biophys J., **85**, 5-15 (2003).

[17] M. Maghrebi, Y. Kantor and M. Kardar, Europhys. Lett. **96**, 66002 (2011).

[18] M. F. Maghrebi, Y. Kantor and M. Kardar, Phys. Rev E **86**, 061801 (2012).

[19] S. Nagai, K. Dubrana, M. Tsai-Pflugfelder, M. B. Davidson, T. M. Roberts, G. W. Brown, E. Varela, F. Hediger, S. M. Gasser and N. J. Krogan, Science **322**, 597-602 (2008).

[20] J. S. Verdaasdonk, P. A. Vasquez, R. M. Barry, T. Barry, S. Goodwin, M. G. Forest and K. Bloom, Mol. Cell **52**, 819831 (2014).

[21] M. Doi and S. F. Edwards, *The Theory of Polymer Dynamics* (Clarendon Press, Oxford, 1986).

[22] B. Albert, J. Mathon, A. Shukla, H. Saad, C. Normand, I. Lger-Silvestre, D. Villa, A. Kamgoue, J. Mozziconacci, H. Wong, C. Zimmer, P. Bhargava, A. Bancaud and O. Gadal, J Cell Biol. **202**, 201-210 (2013).

[23] A. Amitai and D. Holcman, Phys. Rev. Lett. **110**, 248105 (2013).

[24] A. Amitai and D. Holcman, Phys. Rev E **88**, 052604 (2013).

[25] A. Taddei, F. Hediger, F. R. Neumann, C. Bauer and S. M. Gasser , EMBO J **23**, 1301-12 (2004).

[26] Z. Schuss, *Diffusion and Stochastic Processes. An Analytical Approach*, (Springer-Verlag, New York, NY, 2009).

[27] M. Lindner, G. Nir, A. Vivante, I. T. Young, Y. Garini, Phys. Rev. E **87**, 022716 (2013).

[28] N. Hoze, D. Nair, E. Hosy, C. Sieben, S. Manley, A. Herrmann, JB. Sibarita, D. Choquet and D. Holcman, Proc Natl Acad Sci U S A **109**, 17052-17057 (2012).

[29] W. Marshall, A. Straight, J. Marko, J. Swedlow, A. Dernburg, A. Belmont, A. Murray, D. Agard and J. Sedat, Curr Biol. **7**, 930 (1997).

[30] J. Jeon and R. Metzler, Phys. Rev. E **81**, 021103 (2010).

[31] A. Javer, Z. Long, E. Nugent, M. Grisi, K. Siriwatwetchakul, K. D. Dorfman, P. Cicuta and M. C. Lagomarsino, Nat. comm. **4**, 3003 (2013).

[32] C. A. Schneider, W. S. Rasband and K. W. Eliceiri, Nat. Methods **9**, 671-675 (2012).

[33] I. F. Sbalzarini and P. Koumoutsakos, J Struct Biol. **151**, 182-195 (2005).

[34] R. A. Usmani, Comput. Math. Appl. **27**, 59-66 (1994).
